# System Thinking and Citizen Participation Is Still Missing in One Health Initiatives – Lessons From Fifteen Evaluations

**DOI:** 10.3389/fpubh.2021.653398

**Published:** 2021-06-04

**Authors:** Martin Hitziger, John Berezowski, Salome Dürr, Laura C. Falzon, Monique Léchenne, Kennedy Lushasi, Tigran Markosyan, Céline Mbilo, Kelvin N. Momanyi, Ranya Özçelik, Nambiar Prejit, Jakob Zinsstag, Simon R. Rüegg

**Affiliations:** ^1^Section of Epidemiology, Vetsuisse Faculty, University of Zürich, Zurich, Switzerland; ^2^Veterinary Public Health Institute, Vetsuisse Faculty, University of Bern, Bern, Switzerland; ^3^Institute of Infection, Veterinary and Ecological Sciences, University of Liverpool, Liverpool, United Kingdom; ^4^International Livestock Research Institute, Nairobi, Kenya; ^5^Department of Epidemiology and Public Health, Swiss Tropical and Public Health Institute, Basel, Switzerland; ^6^Department of Public Health, Medical Faculty, University of Basel, Basel, Switzerland; ^7^Department of Environmental Health and Ecological Sciences, Ifakara Health Institute, Dar es Salaam, Tanzania; ^8^Scientific Center for Risk Assessment and Analysis in Food Safety Area, Yerevan, Armenia; ^9^Centre for One Health Education, Advocacy, Research and Training, Kerala Veterinary and Animal Sciences University, Wayanad, India

**Keywords:** evaluation, social determinants of health, One Health, disease surveillance, governance, project life cycle, knowledge integration, transdisciplinarity

## Abstract

Tackling complex public health challenges requires integrated approaches to health, such as One Health (OH). A key element of these approaches is the integration of knowledge across sectors, disciplines and stakeholders. It is not yet clear which elements of knowledge integration need endorsement to achieve best outcomes. This paper assesses 15 OH initiatives in 16 African, Asian and European countries to identify opportunities to improve knowledge integration and to investigate geographic influences on knowledge integration capacities. Two related evaluation tools, both relying on semi-quantitative questionnaires, were applied to two sets of case studies. In one tool, the questions relate to operations and infrastructure, while the other assigns questions to the three phases of “design,” “implementation,” and “evaluation” of the project life cycle. In both, the question scores are aggregated using medians. For analysis, extreme values were identified to highlight strengths and weaknesses. Seven initiatives were assessed by a single evaluator external to the initiative, and the other eight initiatives were jointly assessed by several internal and external evaluators. The knowledge integration capacity was greatest during the project implementation stage, and lowest during the evaluation stage. The main weaknesses pointing towards concrete potential for improvement were identified to be a lack of consideration of systemic characteristics, missing engagement of external stakeholders and poor bridging of knowledge, amplified by the absence of opportunities to learn and evolve in a collective process. Most users were unfamiliar with the systems approach to evaluation and found the use of the tools challenging, but they appreciated the new perspective and saw benefits in the ensuing reflections. We conclude that systems thinking and associated practises for OH require not only specific education in OH core competencies, but also methodological and institutional measures to endorse broad participation. To facilitate meta-analyses and generic improvement of integrated approaches to health we suggest including knowledge integration processes as elements to report according to the COHERE guidelines.

## Background

Integrated approaches to health are designed to tackle complex health challenges and exist under different names (1). One Health (OH) is such an approach; it addresses challenges at the interface between people, animals, plants, and their environments, and consequently requires transdisciplinary collaboration among multiple stakeholders from different sectors (2). A key element of the OH approach is the integration of knowledge across sectors, disciplines and stakeholders (3), and at global scale, health innovation can be seen as a result of successful knowledge integration across continental boundaries (4). However, it is not yet clear what form and degree of knowledge integration is needed to conduct successful OH initiatives. Frankson and colleagues collated skills and competences of relevance to OH implementation (5). Other authors have proposed multi-criteria decision analyses, transdisciplinary and participatory approaches, and systems thinking as rigorous methodological tools to support knowledge integration in policy cycles, and have provided case studies of their application (6–9). With the Checklist for One Health Epidemiological Reporting of Evidence (COHERE) a benchmark of reportable elements was stated that should promote the integration of knowledge from the domains of humans, animals and their environment (10). In another attempt, the EU COST Action (TD1404) “Network for Evaluation of One Health (NEOH)” characterised OH and proposed a framework to assess the added value of integrated approaches by comparing knowledge integration (assessed with a specific tool) to the outcomes achieved or expected from the theory of change of a given initiative (11, 12). A method for evaluating knowledge integration capacity in multi-stakeholder governance (EVOLvINC) was derived from the NEOH tool in collaboration with experts from transdisciplinary research, sustainability sciences, and international development research (13, 14).

Structural and procedural barriers to knowledge integration have only recently received increasing attention, mostly in the sustainability sciences, where such barriers are analysed in the context of challenges to the implementation of different stages of transdisciplinary collaborations (15, 16). As far as OH is concerned, underlying epistemological, institutional, political and social factors that are associated with the implementation of multi-sectoral and transdisciplinary approaches appear to be neglected (17, 18). Context also matters, as OH is implemented within a global health framework that is characterised by fragmentation of interests, programs and sectors, a lack of societal participation, and professional focus on very limited areas of expertise (19, 20).

In order to identify a trajectory for improvement and to provide data for future benchmarking, this manuscript analyses empirical evidence of 15 evaluations addressing four research questions:

- Are there particularly strong/weak aspects that point towards opportunities to systematically improve knowledge integration in One Health?
- Can we discern patterns of knowledge integration capacities in relation to geography?
- Do evaluation procedures influence the assessment outcomes?
- Do people working on these initiatives perceive the tools to be understandable and beneficial?

## Methods

This manuscript considers the Standards for Reporting Qualitative Research (21). The utilised evaluation tools were co-created by groups of experts in the context of a systems approach with the assumption of a constructivist epistemology. Three authors contributed to the development of the tools (MH, JZ, SR), while all other authors were members of evaluated initiatives to warrant credibility of the conclusions.

### Recruitment of Initiatives and Evaluation Context

The data was drawn from two groups of initiatives that self-declared using a One Health approach: one group was recruited as case studies to develop an evaluation framework in the EU COST Action NEOH; the other was recruited to test the EVOLvINC tool. Both study groups were convenience samples.

The group of NEOH case studies (H-O) comprised eight previously published evaluations of OH initiatives in Europe and Africa (Table 1). Since they were conducted during the development of the NEOH tool for assessment of One Health-ness (see “evaluation tools, procedures and data processing”), processes varied slightly, and different evaluators carried out the evaluations. Four evaluations were formative, conducted during the implementation stage of the assessed initiative, two were retrospective, and one was prospective. Five used interviews for data collection, three relied partially on document analysis, and two administered questionnaire surveys. Each initiative was scored by two to six evaluators, three were self-evaluations by the initiative participants, and all included external or internal review of the evaluation scores.

**Table 1:** Overview of the eight case studies evaluated with the NEOH tool.

**ID**	**Evaluated initiative**	**Evaluation process**	**References**
H	Brucellosis control in Malta and Serbia.	Retrospective and comparative. 15 interviews and document analysis. Scored by focus group of 6 evaluators from both countries.	(11, 22–24)
I	Cysticercosis surveillance in Portugal.	Prospective self-evaluation by 3 internal evaluators, reviewed by 3 external evaluators.	(12)
J	Southern African Centre for Infectious Disease Surveillance (SACIDS, in five countries of the Southern African Development Community).	Formative, 9 years after inception of the centre. Document analysis, group and individual interviews, and online survey. Scoring by 2 evaluators, who resolved disagreements in discussion. Review by external evaluators.	(13)
K	Animal Health Measures for Control of Cattle Ticks and Tick-Borne Diseases in Southern Zambia.	Retrospective, 25 years after initiative conclusion. Document analysis, and witness interviews. Scoring by external and internal evaluators.	(14)
L	University of Copenhagen Research Centre for Control of Antibiotic Resistance, Denmark.	Formative. Document analysis, semi-structured interviews with 18 project participants, and stakeholder survey. Scoring by 2 internal and 2 external evaluators, validation at the centre's annual event.	(15)
M	West Nile Virus Surveillance, 3 regions of Northern Italy.	Formative, several years after the initiative's inception. Interviews and questionnaires with involved actors. Scoring by 3 internal and external evaluators who resolved disagreements in discussion.	(16)
N	Obesity in European Dogs and Dog-Owners, Europe.	Formative self-evaluation, by 3 initiative participants. Online questionnaire with 20 questions to 24 participants of the initiative. Informal information exchange by mail and face-to face.	(17)
O	Animal Welfare Centre Skopje, Macedonia.	Formative self-evaluation, after 7 years of ongoing work. Scores averaged from 2 evaluators.	(18)

*Please see the citations for more details on each initiative*.

The group of EVOLvINC case studies consisted of six initiatives (A–F) in Africa, Asia, and Europe that responded to a call for collaboration during the 2016 annual conference of the International Society for Disease Surveillance (ISDS). A further study (G) was recruited through personal relations of the authors. Their format varied from individual, small-scale projects to long-term, government-sponsored institutional programs. Six evaluations were formative, and one was prospective. All evaluations were conducted by the same person (MH) using the EVOLvINC tool (see “evaluation tools, procedures, and data processing”). Two initiatives were assessed on-site and five were assessed off-site. Table 2 describes these initiatives and evaluations in more detail.

**Table 2:** Descriptions of initiatives evaluated with the EVOLvINC tool.

**ID**	**State, institutional form**	**Focus, partners, objectives**	**Evaluation process**
A	Republic of Chad. Long-term research-policy program, led by the Swiss Tropical and Public Health Institute (Switzerland), Institut de recherche en élevage pour le développement, and Centre de Support de Santé Internationale (Chad).	Focus is on rabies control in N'Djamena (Chad). Collaboration with governmental institutions on permanent basis. Inclusion of general public and animal owners for specific components. Collaboration with other international research and public health institutes in particular project phases. The current research phase aims at eliminating rabies in dogs, which requires animal surveillance and vaccination, incidence reduction, access to post-exposure prophylaxis, increased population awareness and capacity building for veterinarians and public health experts.	Formative. Several in-depth interviews, field visit, seminar, discussion of evaluation outcomes.
B	Republic of India. Long-term academic research-policy program, which was established in response to a 2015 Kyasanur Forest disease outbreak, and the collaborative investigating protocol was initiated by the Centre for One Health Advocacy, Research and Training (COHEART) at the Veterinary and Animal Sciences University, Kerala.	Focus on surveillance of tick-borne Kyasanur Forest Disease (KFD) in human and monkeys to detect, treat and reduce infection risk in Wayanad, Kerala (India). It builds on a cross-sectorial and transdisciplinary collaboration between human and animal health institutions, and forest departments at local, regional and national levels, including affected tribal populations. Objectives are to institutionalise a One Health Surveillance mechanism by linking stakeholders, to reduce prevalence and abundance of vectors, to reduce disease incidence in humans and monkeys, to build awareness and to induce behavioural change.	Formative. In-depth interview, field visit, seminar, stakeholder/actor questionnaire, discussion of evaluation outcomes.
C	Republic of Armenia. Governmental surveillance program, led by the Veterinary State Authority in the Ministry of Agriculture.	Focus is to enable a coordination mechanism for zoonotic disease surveillance. It implements the national brucellosis strategy on behalf of the government, coordinates between the ministry of agriculture, farmers, community veterinarians, public health and environmental sectors through official channels. Its objectives are to enable a National strategy for brucellosis control for human and livestock based on risk assessment and better data-sharing through adequate legislation and IT systems.	Formative. In-depth interview, discussion of evaluation outcomes.
D	Congo-Central. Collaborative research project, led by the Swiss Tropical and Public Health Institute.	Focus on rabies surveillance in 6 savannah communities. Collaboration with a local university, labs, schools, hunters, nurses, and national ministries of health, agriculture and animal health. Its objectives are to elicit rabies incidence in humans and animals, phylogenetic analysis of the virus, to sensitise the population, and to analyze dog demographics.	Formative. In-depth interview, discussion of evaluation outcomes.
E	Republic of Kenya. Long-term research-policy program, led by the Institute of Infection, Veterinary and Ecological Sciences (University of Liverpool, UK), and the International Livestock Research Institute (Nairobi, Kenya).	Focus on zoonoses in livestock and humans in western Kenya (ZooLinK). Partners from grass-root to policy level from academic, health and policy sectors. Its objectives are to assess the burden of disease and incidence patterns of various zoonotic diseases in animals and humans, to understand seasonal changes, and to instal and evaluate a cost-effective, and sustainable surveillance system.	Formative. In-depth interview, discussion of evaluation outcomes.
F	United Republic of Tanzania. Long-term research-policy program, led by the Ifakara Health Institute (United Republic of Tanzania), and the Nelson Mandela African Institute of Science and Technology (Univ. of Glasgow, UK).	Focus on rabies elimination. Partners are the national ministries of health, livestock, natural resources and tourism, several research and pharmaceutic industry institutions, the World Organisation for Animal Health (OIE), community members, the World Health Organisations country office, social scientists, and local leaders. Its objectives are to conduct vaccine trials in areas without infrastructure, to test strategies that minimise risk of re-introduction, to improve implementation guides for surveillance and control, to understand barriers to rabies elimination, to improve access and administration of human post exposure prophylaxes to bite victims, and to build a precise and transferable surveillance system.	Formative. In-depth interview, discussion of evaluation outcomes.
G	Switzerland and the Republic of Chad. Collaborative research sub-project of a larger initiative, in the process of establishment. Led by the Veterinary Public Health Institute (University of Bern, Switzerland) and the Institut de recherche en élevage pour le développement, and Centre de Support de Santé Internationale (Chad).	Establishment and evaluation of community based surveillance systems in Switzerland and Chad. Collaboration with networks of governmental and non-governmental organisations, animal holder groups, and academic/lab-analytic partner institutions in both countries, plus IT companies for setting up surveillance and communication infrastructures. Compares the two syndromic surveillance systems with regard to tool acceptance, sensitivity, timeliness, early detection, and advantages of combined human/animal reports (latter in Chad only).	Prospective. In-depth interview, seminar, discussion of evaluation outcomes.

*The evaluation processes are further detailed in the Methods section, but specified here for reference*.

### Evaluation Tools, Procedures, and Data Processing

Two evaluation tools were employed: a) the NEOH tool assesses the “One Health-ness” of initiatives with a catalogue of ~80 semi-quantitative questions about knowledge integration (12). The tool was later scrutinised in a more generic context. The second tool b) was developed in collaboration with experts from transdisciplinary research, sustainability sciences and international development research: Evaluating knOwLedge Integration Capacity in multi-stakeholder governance (EVOLvINC) (13).

First, the case studies from the NEOH group (H-O) were used to test the NEOH tool and all employed the concept of mapping the system in which the initiative is situated and then use these data to answer 80 questions relating to six aspects: systemic thinking, planning, transdisciplinary working, sharing, learning, and the systemic organisation (14). The framing of these aspects was established in an earlier workshop to characterise OH (11). Ultimately, the semi-quantitative scores of the questions between 0 and 1 provide a median score for each aspect that is then represented as a spoke in a hexagonal spider diagram. The surface of this diagram is interpreted as the OH index. The surface spanned by the first three aspects, divided by the surface of the latter three aspects, is the OH ratio (14). These evaluations were conducted by various evaluators in parallel to developing the evaluation method itself.

**Figure 1:**
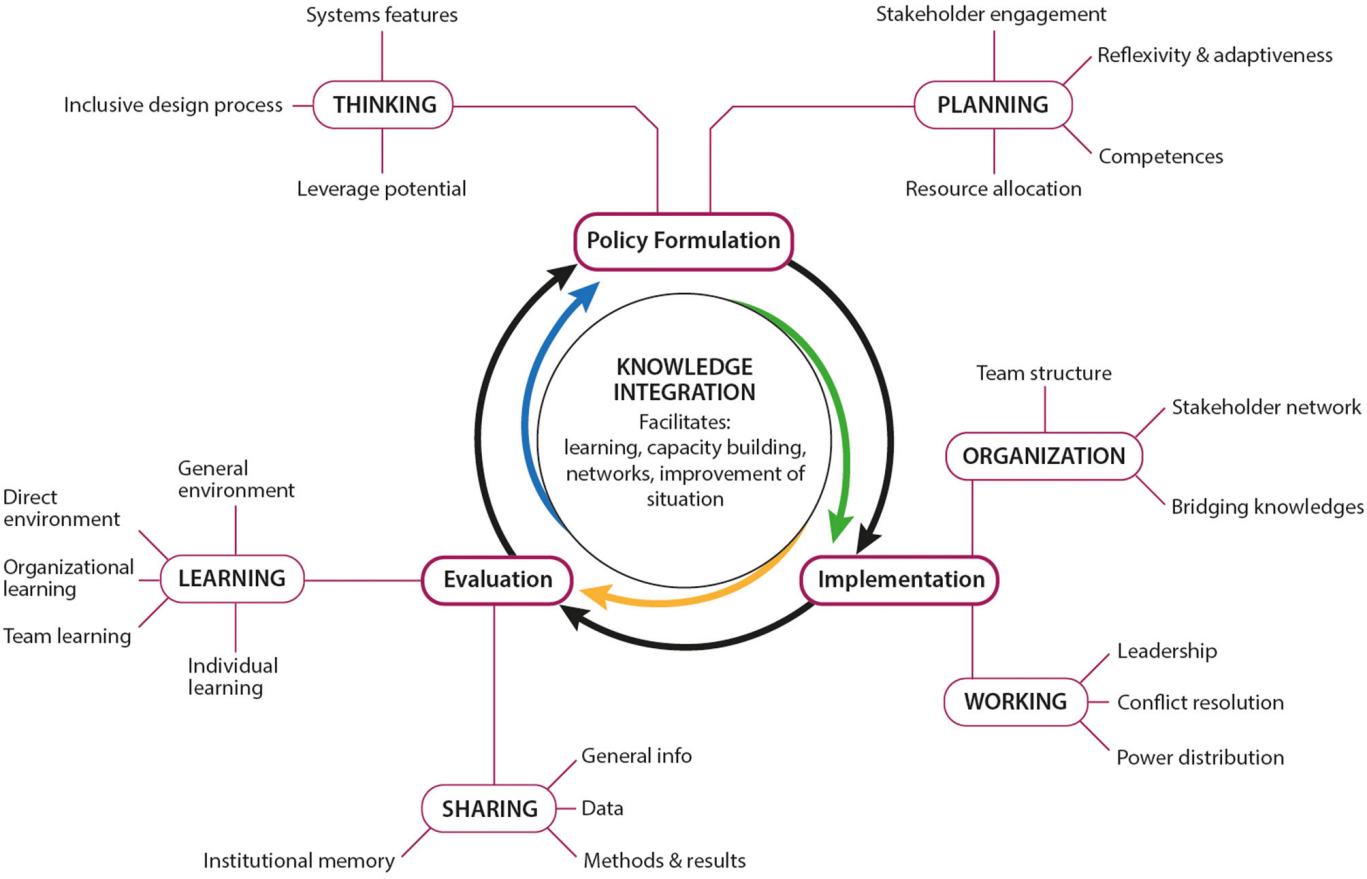
Structure of the EVOLvINC tool for Evaluating knOwLedge Integration Capacity in multi-stakeholder governance according to Hitziger et al. (13). The bold black cycle contains the three stages of the project life cycle (formulation, implementation, and evaluation). The knowledge integration capacity in each of these stages is assessed by two aspects (bold, capital letters), and each aspect is measured by 3–5 criteria. The questions attributed to each criterion in the EVOLvINC tool are stated in Table 3.

The EVOLvINC tool arranges the same six aspects used in the NEOH tool along the three stages of the project life cycle (13), but the aspects are operationalized in 3–5 criteria (Figure 1). Each criterion is measured by 3–5 indicators articulated as questions that are scored on a four-level Likert scale. The levels translate into a score between 0 (not conducive to knowledge integration) and 1 (highly conducive to knowledge integration). The median indicator scores correspond to the criterion score, and median criteria scores are aggregated to aspect scores. The comparison of the questions used in the two tools is provided in Table 3.

**Table 3:** Comparison of the NEOH tool for assessment of “One Health-ness” and the EVOLvINC tool.

	**EVOLvINC criterion**	**EVOLvINC question**	**NEOH question**
Thinking	Inclusive design process	How are objectives and their relative importance established?	
		Has a theory of change been elaborated to match the objectives of the initiative?	To what degree does the initiative identify subsystems and interactions between them and integrate this structure in the theory of change?
		How do the objectives and the theory of change reflect multiple perspectives, value systems and beliefs?	To what degree does the initiative consider the beliefs about evidence, values about health, cultural grounding as factors affecting the theory of change?
		How do the methods, scales and criteria of success reflect multiple perspectives, value systems and beliefs?	How well do the number of dimensions and scales reflect an integrated approach to health?
			How well does the consideration of dimensions by the initiative match the dimensions of the system in which it operates (context)?
			How balanced is the consideration of the different dimensions by the initiative?
			How many scales are considered in the different dimensions of the initiative?
	Consideration of systems features	Is the problem that the initiative addresses an event, a pattern, or a structure?	
		How are time delays between different processes in the system considered?	Are time delays recognised in the theory of change?
		How are feedback loops and causal interactions in the system considered?	How are the dynamic feedback loops of the system identified?
	Leverage potential	Does the initiative comprehensively translate the problem into scientific or developmental questions?	Is the One Health challenge adequately translated into scientific or developmental questions? [Organisation]
			What feature of the system is targeted by the initiative?
		Where is the initiative situated in relation to the chain of events causing the problem?	Where is the initiative situated in relation to the chain of events causing the problem and responding to it?
	Integrated Approach to Health, Environment, Sustainability		How well does the initiative consider One Health and the three pillars of sustainability?
		How well does the initiative match its environment?	How well does the initiative match its environment?
			Are time delays recognised in the theory of change?
Planning	Identification and engagement of sectors, actors, and stakeholders	How are sectors and disciplines identified, that affect or are affected by the problem that the initiative targets and are thus relevant for achieving its objectives and for leveraging impact?	Stakeholder identification process ('the right people heard'?)
			Actor identification process (‘the right people involved'?)
		How is stakeholder commitment assured?	Planning of engagement of stakeholders, use of stakeholder input and effect on stakeholder perceptions
			Planned organisation needed to reach common aim(s)?
			Common aim(s) in initiative
	Reflexivity and adaptiveness	Which opportunities for reflection and self-assessment does the initiative provide?	How appropriate is the time and budget allocated for self-assessment?
		How flexible is the project execution and timeline to respond to internal or external changes in the short term (<= 1 year)?	How flexible is the project design and timeline to respond to internal or external changes at short-term? [Working]
		How flexible is the project execution and timeline to respond to internal or external changes in the mid term (1–3 years)?	How flexible is the project design and timeline to respond to internal or external changes at mid-term? [Working]
		How flexible is the project execution and timeline to respond to internal or external changes in the long term (≥3 years)?	How flexible is the project design and timeline to respond to internal or external changes at long-term? [Working]
			Is the initiative built on an iterative process?
	Competences	How adequate are the competences of team members and actors to achieve the objectives?	Are the competences displayed by the various disciplines appropriate to the problem and its solution (relevant knowledge applied, roles in the initiative, possibilities for implementing results)? [Organisation]
		How adequate are the methods to achieve the objectives?	
	Resource allocation	How adequate are the budget allowances to achieve the objectives?	
		How adequate are the time allowances to achieve the objectives?	
			For objectives 1–10: Planning of ressource allocation for specific objectives directed towards One Health outcomes: Specific objective X
Organisation	Internal team structure	If more than one team is present, how are the inter-team relations?	If more teams than one are mentioned, how good are the inter-team relations?
		How are the team objectives established?	Do all teams have clear objectives?
		How are individual roles established and differentiated?	How clearly are the roles differentiated for team members within the team?
			How many teams are present in the initiative?
			How is teamwork implemented in this initiative?
			Are/were the teams recognised by the community/department/s/official organisations as clearly defined team(s)?
			How closely do team members work together to achieve the teams' objectives?
			How frequently do(es)/did the team(s) meet to discuss their effectiveness and how it could be improved?
			How well do/did the disciplinary composition and the competence in the team(s) permit the working towards the essential aspects of their objectives?
	External stakeholder network	How frequently are stakeholders involved in the initiative?	To what extent is/was the non-scientific community involved during the execution of the initiative? [Working]
			To what extent is/was cross-sectorial involvement present during the execution of the initiative? [Working]
		How intense is the collaboration between stakeholders in the initiative?	To what extent do/did the different disciplines work together? [Working]
	Bridging knowledges	Which methods are used to ‘bridge', ‘link' or ‘integrate' the knowledge of team members, actors and stakeholders?	Did the initiative provide relevant innovation in relation to the state of knowledge and the OH challenge?
		Which processes are used to ‘bridge', ‘link' or ‘integrate' the knowledge of team members, actors and stakeholders?	
			Is the current state of knowledge taken into consideration (including information about relevant societal issues and structures)?
Working	Power distribution	How is the distribution of power or influence between team members and stakeholders from different (i) Disciplines?	Are/were there power (i.e., academic or disciplinary dominance) or gender imbalances within the initiative, which risk biassing the process? (i) Across disciplines
		(ii) Sectors?	(ii) Across sectors
		(iii) Ethnicities?	(iii) Across ethnicities
		(iv) Social classes?	(iv) Across social classes
		(v) Genders?	(v) Across gender
		(vi) Cultures?	(vi) Cultural issues
		(vii) Religions?	(vii) Religious issues
	Leadership	Is the management structure appropriate to support the team and actors in achieving the initiative's objectives?	How well do the management structures match and support the initiative's goal and combination of disciplines and fields of expertise? [Organisation]
		How would you characterise the leadership approach to project management?	How would you characterise the leadership in the initiative in regard to task-orientation, relationship-orientation and change-orientation? [Organisation]
		How open-minded is the leadership to creative input?	Does the initiative demonstrate open mindedness? [Organisation]
		How flexible are internal hierarchies and decision making in adapting to circumstances and tasks?	Does the initiative demonstrate changing hierarchies? [Organisation]
	Conflict resolution	How does the leadership manage conflicts and tensions?	Does the initiative demonstrate ability to bear and manage tensions? [Organisation]
		At what level are conflicts resolved?	
		How does the team react to conflict?	
			Is transdisciplinarity required to solve this problem? And what are the benefits of using a transdisciplinary approach in the initiative rather than conventional/disciplinary approaches?
			How diverse are/were the disciplines, methods, scales of analysis and/or social actors involved? Enumerate all disciplines, methods, dimensions and scales of analysis considered, as well as the social actors involved, as they were introduced in the OH Thinking.
			How likely is reflection going to feed back into corrective action within the initiative?
			How is interaction between people organised to foster collaboration across the initiative?
Sharing	Process for information exchange	How adequate are resources allocated to ensure information sharing?	Have resources been allocated to facilitate and ensure necessary data and information sharing?
		How are agreements concerning information sharing established?	Have appropriate (e.g., formal/written/signed) agreements been made concerning data sharing in the initiative? How well/how much are data being shared between people within the initiative?
		Does the initiative have internal mechanisms to facilitate the exchange of information within the initiative and are these used?	Does the initiative have appropriate mechanisms in place to facilite sharing of information within the initiative and are these used? (e.g., newsletters, workshops, reports available to all, results getting published, online information sharing platform….)
		Does the initiative have external mechanisms to facilitate the exchange of information within the initiative and are these used?	Does the initiative have appropriate mechanisms in place to facilite sharing of information outside the initiative and are these used? (e.g., newsletters, workshops, seminars/conferences, reports available to all, results getting published, online information sharing platform….)
	Data	How adequate are procedures to ensure the quality of shared data?	Are mechanisms/procedures in place to ensure data quality to allow sharing, e.g., data completeness, error-checking and correction of errors, clear and accurate descriptions of variables and of aggregations/calculations, documentation available.
		How adequate are procedures to ensure safe and appropriate data storage and accessibility?	Are mechanisms/procedures in place to ensure safe and appropriate data storage (e.g., type of software, server, backup) with safe accessiblity to facilitate sharing? (e.g., is extraction of data feasible without access without data managers, or are expert managers readily available for extraction of data, is the process of data extraction bureaucratic/ cumbersome/overly time-consuming?)
		How well/how much are data shared within the initiative?	
	Methods and results	How well/much are methods shared between people within the initiative?	How well are methods shared between people within the initiative?
		How much/well are results shared between people within the initiative?	How are results shared between people within the initiative?
	Institutional memory	Does the initiative create or use long-term institutional knowledge reservoirs for data, methods and results?	How well does the initiative include the creation or use of potential institutional knowledge reservoirs for data, methods and/or results over time? For instance publications, detailed reports/manuals, database descriptions, standard operating procedures, introductions to inform new staff on essential procedures etc.
		Are procedures in place to safe-guard access to data, information and results in case of system change?	Are mechanisms/procedures in place to safe-guard access to data, information and results in case of system change, e.g., change of IT-system, data ownership, institutional organisations.
Learning	Individual learning	How often do individuals receive information which may be understood and may potentially lead to learning, but it is not put intp practise in or outside the initiative by the individuals (basic learning)?	Individual: Basic learning
		How often is the information understood, learnt and applied to improve procedures, competences, technologies and paradigms without challenging the individuals underlying beliefs and assumptions (adaptive learning)?	Individual: Adaptive Learning
		How often is the information understood and learnt by individuals and applied to improve procedures, competences, teachnologies and paradigms as a result of modified underlying beliefs and norms of individuals (generative learning)?	Individual: Generative Learning
	Team learning	How often do teams meet to exchange information for reporting purposes (basic learning)?	Team: Basic learning
		When teams meet, how often are different views presented, defended and discussed to find the best possible view to support decision making (adaptive learning)?	Team: Adaptive Learning
		When teams meet, how often are complex issues explored through dissection of views and assumptions of team members resulting in a move towards building new ideas, views or approaches (generative learning)?	Team: Generative Learning
	Organisational learning	How often is existing/circulating information and knowledge collected and stored (basic learning)?	Organisational: Basic Learning
		How often is collected information shared, discussed and acted upon at various levels within the organisation (adaptive learning)?	Organisational: Adaptive Learning
		How often is collected information shared, discussed and leads to change in fundamentals and objectives across all levels within the organisation (generative learning)?	Organisational: Generative Learning
	Direct environment	How often is the direct environment of the initiative (involved stakeholders) supportive for adaptive learning?	Direct environment: For Adaptive learning
		How often is the direct environment of the initiative (involved stakeholders) supportive for generative learning?	Direct environment: For Generative learning
	General environment	How often is the general environment of the initiative (e.g., culture, economics, political situation) supportive for adaptive learning?	General Environment: For adaptive learning
		How often is the general environment of the initiative (e.g., culture, economics, political situation) supportive for generative learning?	General Environment: For Generative learning

*Questions highlighted in light and dark grey are attributed to the organisation and working aspects in the NEOH tool, respectively*.

The EVOLvINC tool was applied in a three-stage process:

1. To build a common ground, information on the conceptual background of the evaluation and the complete EVOLvINC questionnaire (Supplementary Material) were provided to the initiative leaders and discussed with them. For on-site evaluations, these aspects were presented during a seminar. In initiative B, this seminar was a certified full day event attended by 150 stakeholders and students.
2. EVOLvINC was administered in structured interviews with initiative leaders and participants. Each took ~3 h, allowing sufficient time to address problems of understanding, rephrase questions where required, and discuss how the answers translated to the four-level Likert scale (see below). For on-site evaluations, interviews were complemented with discussions with other initiative participants.
3. The results of the analysis were provided to the initiatives in an executive summary and in graphical form. They were discussed and opportunities for improving the initiative's capacity to foster knowledge integration were elaborated with the leadership.

The tools provide two different perspectives using similar data: while the NEOH tool compares operational aspects (working, thinking, planning) and infrastructural aspects (learning, sharing, organisation) in a OH ratio, EVOLvINC links the aspects to the project life cycle. Thus, the NEOH representation facilitates reflection about investments in the system operations or infrastructure, and the EVOLvINC tool conveys the contribution of these aspects to the project life cycle and the capacity to evolve. In the EVOLvINC tool some questions are deleted and added compared to the NEOH tool. Also, a number of questions are attributed to different aspects in the two tools (Table 3). Questions related to reflexivity and adaptiveness in the working aspect of the NEOH tool are attributed to the planning aspect in EVOLvINC. The involvement of external stakeholders in the working aspect of the NEOH tool is attributed to the organisation aspect of the EVOLvINC tool. Questions probing for leadership are in the organisation aspect of the NEOH tool, but in the working aspect of EVOLvINC. It is thus not possible to compute the OH index and ratio relying on the median of the aspects, but imperative to aggregate the scores of the relevant questions. As studies A-G were collected based on the data requirements of the EVOLvINC tool, while studies H-O relied on the NEOH tool, availability of data was not identical. However, as this concerned to only a small proportion of questions, missing and surplus questions were simply omitted for aggregating calculations. We deemed this appropriate as the main aggregating operation is the median, which is relatively robust in such conditions. For the NEOH case study from North Macedonia, detailed data to compute the EVOLvINC aggregates was not available.

For both tools, the aggregated numerical results are complemented by a qualitative synthesis and recommendations for improving the initiative's capacity to foster knowledge integration. Due to their different composition, the aspects “working,” “planning,” and “organisation” are discussed differently in the context of either tool.

Since the number of studies does not allow for statistical analysis, the comparisons are presented as coloured tables with light colours for the scores in the lowest quartile and dark for scores in highest quartile. To complement, we computed the median of the aspects for each initiative and then compared medians of different clusters of initiatives in terms of median, OH index and OH ratio. With these representations we investigate whether there is a difference in scores obtained from internal and external evaluations, and if we could observe a difference across the geographical regions in which the case studies were situated. Furthermore, this helped us to identify particularly strong/weak aspects that point towards opportunities to systematically improve knowledge integration in OH.

### User Satisfaction

Care was taken to assess the usability and potential adoption of the evaluation tools by OH practitioners. The NEOH case studies contained a critical appraisal of the tool as part of the discussions in the published manuscripts. For the ISDS case studies, a final discussion with the initiative leaders allowed us to gather structured feedback with regard to the lessons they learnt throughout the assessment, and their perspectives on the relevance and applicability of the tool and the evaluation process. In the present manuscript, we combine these qualitative results with observations drawn from the visual analysis of the scoring exercise to determine whether initiatives perceived the tools to be understandable and beneficial, and to determine whether there were indicators on how knowledge integration processes in OH can be systematically enhanced.

## Results

The scores of the aspects ranged from 0.18 to 1 (Table 4). The table is colour coded to highlight the top (dark) and lowest (light) quartile of the aspect scores. It illustrates that sharing and learning are the least developed aspects, both linked to the evaluation stage of the policy or project life cycle. The strongest scores were achieved for the thinking and organisation aspects. The OH indices ranged from 0.29 to 0.74 with a median of 0.36, while the OH ratios were between 0.79 and 1.97 with a median of 1.22 (Table 5). All OH ratios were larger than one except for those of three initiatives.

**Table 4:** Aspect scores computed for the 15 case studies using the EVOLvINC tool.

	**ID**	**Thinking**	**Planning**	**Organisation**	**Working**	**Sharing**	**Learning**	**Median**
Chad	A^*^	0.83	0.75	0.83	1.00	0.66	0.66	0.79
Southern Africa	J	0.53	0.60	1.00	0.60	0.18	0.69	0.60
Zambia	K	0.65	0.60	0.55	0.60	0.45	0.50	0.58
Congo	D^*^	0.50	0.58	0.67	0.66	0.50	0.33	0.54
Kenya	E^*^	0.50	0.79	1.00	1.00	0.83	0.66	0.81
Tanzania	F^*^	1.00	0.92	0.83	0.66	0.66	0.66	0.75
Chad	G^*^	0.66	0.75	0.66	0.71	0.83	N/A	0.71
India	B^*^	0.83	0.58	1.00	0.83	0.50	0.33	0.71
Armenia	C^*^	0.33	0.75	0.50	0.66	0.75	0.66	0.66
Malta	H	0.70	0.60	0.80	0.60	0.55	0.65	0.63
Serbia		0.65	0.70	0.70	0.65	0.70	0.60	0.68
Portugal	I	0.78	0.85	0.60	0.50	0.48	0.50	0.55
Denmark	L	0.60	0.48	0.60	0.60	0.53	0.48	0.56
Italy	M	0.80	0.88	0.75	0.58	0.84	0.65	0.78
Europe	N	0.70	0.45	0.70	0.90	0.43	0.35	0.58

Data was extracted from the NEOH case studies to aggregate the aspect scores according to the EVOLvINC tool. The top quartile of all scores is dark green and the bottom quartile is light green. Initiatives are grouped geographically, and those evaluated by the same evaluator are indicated with an^*^.

**Table 5:** One Health index and ratio computed according to the NEOH tool.

	**ID**	**OH-index**	**OH-ratio**
Chad	A^*^	0.51	0.79
Southern Africa	J	0.36	1.50
Zambia	K	0.33	1.14
Congo	D^*^	0.29	1.67
Kenya	E^*^	0.70	0.76
Tanzania	F^*^	0.65	1.84
Chad/ Switzerland	G^*^	N/A	N/A
India	B^*^	0.36	1.41
Armenia	C^*^	0.36	0.95
Malta	H	0.54	1.37
Serbia		0.49	1.14
Portugal	I	0.31	1.97
Denmark	L	0.341	1.102
Italy	M	0.646	1.219
Europe	N	0.291	1.097
North Macedonia	O	0.421	1.749

Questions were extracted from the EVOLvINC questionnaire to compute the OH index and ratio, missing questions were omitted. The top quartile of the OH index and ratio is dark brown and the bottom quartile is light brown. Initiatives are grouped geographically, and those evaluated by the same evaluator are indicated with an^*^.

### Opportunities to Improve Knowledge Integration Capacity in One Health

To report the most significant observations from the evaluations of the ISDS case studies (initiatives A–G), we cite some qualitative notes associated with the criteria that had most scores in the top and bottom 10 percentile within the respective project life cycle phase (Supplementary Material, Excel: “Criteria EVOLvINC group” sheet).

#### Knowledge Integration Capacity in Initiative Formulation

The most positively evaluated criteria of the formulation phase were “leverage potential” (B, F), “competences and methods,” (E, F) and “resource allocation” (C, F). For example, initiative F was scored with a high leverage potential, since it addresses rabies in Tanzania with a comprehensive approach that encompasses human and animal health alongside societal and economic dimensions, and since it addresses impacts caused by the disease, by facilitating affordable and accessible rabies vaccinations in remote areas that are epidemiologically affected. Initiative E received high scores for competences and methods, since the project leaders reported field and research teams in Kenya to be trained in elaborate methods and capable in all required professional competences, including human and animal health, laboratory and clinical services, and data analysis. Initiative C received high scores on allocated resources since their governmental supervisors assigned sufficient financial and time allocations to ministry officials to carry out all the duties that are required from them. The weakest criterion was the “consideration of system characteristics” (C–E). For example, initiative E was identified to be working at the level of population patterns of zoonoses in Kenya rather than at the level of transmission structures, and activities during project formulation did not explicitly consider time delays, feedback loops, and causal interactions between different processes.

#### Knowledge Integration Capacity in Initiative Implementation

The most positively evaluated criteria for the implementation phase were “internal team structure” (A, B, D, E, F), “power distribution” (B, E, F, G), and “conflict resolution” (A, D, E) processes. For example, initiative D received high scores since two fieldwork teams in each of the six communities were clearly defined. Even though inter-team relations in each community ranged between support, competition, and ignorance, each team was working towards explicitly defined objectives. The power distribution was generally assessed as equal, but with a high interquartile range (IQR). Highest levels of inequality were recorded for differences between disciplines and sectors, such as medical and veterinary professions. For example, initiative B received high scores for a power distribution despite a lack of participation of local tribal populations, since it achieved a balanced participation of different disciplines and sectors, societal classes and genders (which could be confirmed by the evaluator (MH) during a 1 day seminar event in Kerala). Initiative A was scored high on conflict resolution, since it considered that collaborators were mostly motivated by the mission to eradicate a zoonotic disease (rabies), while financial conflicts were mostly resolved through imposition and personal conflicts through mediation. Teams reported approaching most conflicts through dialogue (though concealment had possibly occurred as well), and conflicts were usually resolved on factual levels or through team building. The poorest scores were achieved for “external actor and stakeholder network” (C, D) and “bridging knowledge” (C, G). For example, initiative C, a government effort, reported that external stakeholders had never been involved or participated. For the criterion on bridging knowledge, a rich set of integration *methods* was employed in many initiatives, while scores for integration *processes* were often low. While using various methods to integrate knowledge (including written information exchange, unstructured dialogues, mediation through bridge persons and boundary institutions, and joint project design), initiative G considered, for example, that its' knowledge integration processes were mostly centralised though the project leader and project management.

#### Knowledge Integration Capacity in Initiative Evaluation

The strongest evaluation scores for the evaluation phase were achieved for the “sharing of methods and results” (C, E, G) and the “direct learning environment” (A, F). For example, initiative G reported sharing its methods and results with the entire initiative, while initiative A reported that workshops demonstrated how involved stakeholders were frequently supportive of adaptive (improving existing procedures), and even generative (questioning existing norms) learning. The weakest criteria were “organisational learning” (A, D) and “general learning environment” (A, B, D). For example, initiative D considered that there was no mechanism for information storage (basic learning) at an organisation level, and while results were discussed within the team in a bi-weekly rhythm, these would never result in generative organisational learning (change in fundamentals and objectives). Initiative B reported that during the 4 years of its existence, its general environment (cultural, economic or political institutions beyond the core initiative stakeholders) had rarely been receptive to either adaptive or generative learning.

### Geographic and Procedural Influences on Evaluation Outcomes

The median score of the ISDS initiatives assessed by the first author was 0.71, whereas of those conducted by different assessors in the NEOH group was 0.59. The median of the OH indices of the ISDS studies was 0.44 and that of the NEOH studies 0.36, while the median OH ratios were 1.18 and 1.22 respectively. Aggregation by geographical situation resulted in a median score of 0.71, 0.68, and 0.60 for initiatives situated in Africa, Asia, and Europe, respectively. The median OH indices were 0.44, 0.36, and 0.42 respectively, while the median OH ratio was 1.32 for Africa, 1.18 for Asia and 1.22 for Europe.

### User Satisfaction

All initiatives appreciated the evaluation process, theoretical approach, and questionnaires. Each initiative experienced the evaluation as a trigger for important reflections that they intend to apply in the future. The lessons learnt were derived from all phases of the evaluation process, and the conceptual background. Insights that were singled out as particularly relevant or thought-provoking included each of the six aspects, and the criteria “inclusive design process,” “identification and engagement of sectors, actors and stakeholders,” “reflectivity,” “internal team structure,” “external stakeholder network,” “power distribution,” “leadership,” and “conflict resolution.” Several initiatives realised the importance of additional systemic and environmental factors of relevance to their OH focus.

Participants from several initiatives mentioned that they required time to understand the process and rationale of the evaluation. Interviews were perceived to be long and challenging, and some concepts were found to be complex and abstract. Questions addressing systems thinking frequently needed additional explanations or adaptation to the concrete context at hand. Some questions required simplification. These findings concur with evaluator (MH) observations and with the conclusions drawn by evaluators of two reviewed studies (I, L). However, the interactive, iterative process and the graphical representations of the EVOLvINC rationale and results were reported to be helpful for participants to attain the necessary levels of abstraction and complexity. The combination of qualitative and quantitative assessments was considered useful for allowing joint scoring by evaluators and participants, based on reflection, richness of detail, and development of personal input by the interviewees (J, L). The two evaluations (A, B) conducted during field visits of the first author highly valued the in-person collaboration.

## Discussion

Our study includes a rich data set with 15 initiatives located in 16 African, Asian, and European countries from two different study groups. Despite being a convenience sample and thus not representative of the total body of activities that self-declare as “One Health initiatives,” they appear to underpin general concerns in regard to such initiatives and also delineate a path for improvement.

### Opportunities to Improve Knowledge Integration in One Health

Knowledge integration seems to be emphasised by most OH initiatives primarily during the implementation phase, but there are opportunities to enhance participation and knowledge integration in all three phases of the project life cycle. In the formulation phase, a strong baseline was provided through the leverage potential and a diversity of competencies. This was endorsed by the attention given to power distribution and conflict resolution during implementation, and the willingness to share data. The main challenges in adopting a systems perspective were rooted in a lack of consideration of systemic characteristics. This is amplified by the lack of external stakeholder engagement, the poor bridging of knowledge in the implementation phase, and the limited attention given to an initiative or policy as a learning organisation with evolutionary features.

These findings support the call for specific education in OH core competencies, and emphasise that systems thinking and associated practises are crucial skills to develop in the OH community (5, 22). This necessity is well-illustrated by Prieto et al. (23), who reported that initiative N did not engage with stakeholders or in any intersectoral collaborative processes. On the contrary, Buttigieg et al. (24) describe the evolutionary process through one century of trial and error to eradicate brucellosis in Malta, culminating in a systemic and inclusive approach. These examples also emphasise the high dependency of OH initiatives on their work environment. Low scores for conduciveness of the general environment to learning reflect the extremely challenging institutional and societal contexts of many such initiatives. Notably, higher learning scores for initiatives that are close to national or regional governments highlight the value of involving high-ranking decision makers. However, the relative ease of mobilising these stakeholders compared to engaging citizens may add to the imbalance and emphasises the necessity to actively mobilise the tacit knowledge held by citizens when embarking on a OH initiative. In addition to involving the appropriate diversity of stakeholders, transforming different bodies of observations into joint narratives of how situations emerge and might unfold in the future requires true collaboration, with dedicated processes for information sharing and bridging, and a context that enables learning. While many methods are employed to bridge and integrate knowledge, these are predominantly focused on a small set of experts or project leaders. Processes that facilitate common group learning and reduce inequality between participating disciplines and sectors are needed to further enhance knowledge integration and strengthen networks for collective action. This concurs with Léger et al. (25), who suggest that lack of true collaboration results in low scores for sharing and learning: “*Although the […] theory of change was highly relevant, reasonably well-planned and highly integrated with many disciplines and with relevant stakeholders involved from the beginning, it proved difficult to carry out the OH approach in practise, and many of the actors went back to unisectorial and disciplinary work in their daily tasks. This […] potentially reduced the societal impact of the initiative*.”

Sharing and learning contribute to the evaluation phase which, on average, got lower scores than the other phases. This observation resonates with the claim that evaluation is a deficit in current practise of OH (26). Consequently, the low scores for generative learning (addressing and revising deep, complex beliefs, assumptions, paradigms, or objectives) provide some insight into why the envisaged paradigm shift through OH has not yet occurred (27).

### Geographic and Procedural Influences on Evaluation Outcomes

The small size of the sample and the geographical bias in the groups make it difficult to attribute further procedural effects to the scoring results with certainty. Yet, a median OH ratio above one is consistent across initiatives from all three continents, which suggests that more emphasis is given to operational aspects than to setting up infrastructure. This finding probably reflects a selection bias, as initiatives setting up infrastructures to share, learn and distribute leadership may not self-identify as “OH initiatives.” Secondly, high scores in “sharing methods and results” within overall low scores in sharing and learning reflect the scientific community through which the studies were selected, and the importance given to publishing methods and results in research.

From a geographical standpoint, case studies located in Africa had a higher median score and IQR (0.71, 0.18) than the European studies (0.60, 0.10). At closer inspection they have higher scores and less variance in the evaluation phase than the European studies. The median OH indices did not differ much (0.44 vs. 0.42), but the median OH ratios (1.32 vs. 1.22) suggest that the African studies put particularly more emphasis on operational aspects and particularly less on infrastructure. We are thus inclined to think that the funding context of the studies located in Africa (primarily international development funding) require more effort for evaluation and that they have less impact on the local infrastructure for knowledge integration, than in the European context where domestic funding requires less accountability and promotes local infrastructure investments.

When comparing the two study groups, the median score and the IQR were higher in the ISDS group (0.71, 0.085), than in the NEOH study group (0.59, 0.067). These differences were most pronounced for the implementation phase and were also reflected in analogous differences in the OH indices. Because of the different evaluation procedures in the two groups, we cannot discern if this indicates a more severe scrutiny in the NEOH study group or whether it is due to the characteristics of the two samples.

### User Satisfaction

All initiatives in this study appreciated the evaluation approach as an opportunity for a learning process and identified specific indicators or elements that they intend to improve or apply in the future. The combination of qualitative and quantitative assessments was deemed beneficial. Acquiring the necessary knowledge to understand and interpret the systems approach was found to be time consuming and challenging. Some concepts were even too abstract for a straightforward application. In these instances, the iterative process and graphical support were considered helpful, and the in-person collaboration was highly appreciated. Several initiatives (D, E, G) suggested further collaborative evaluation workshops were needed to enhance understanding and depth of responses to intellectually challenging or politically sensitive concepts, such as systems thinking, project design, and power distribution. This aligns with other authors who have advocated for the use of focus groups to investigate sensitive issues (28).

A final note should be made regarding the timing of an evaluation within an initiative's life cycle. Both tools are easily adapted to prospective, formative or retrospective evaluations with only minor rephrasing. Fonseca et al. (29) recommend prospective and repeated formative evaluations at early stages of the initiative, since they allow for anticipation of subsequent aspects and enhancement of knowledge integration capacity in the future. This would require a short and concise questionnaire that can be used in self-evaluations without much expertise. In the present study, prospective evaluation of the learning aspect was found challenging (G, I) since many learning opportunities develop spontaneously during implementation stages, and many questions relate to actual lessons learnt, rather than mere opportunities to learn. At the other end of the cycle, retrospective evaluations may be limited by data availability if collaborators are hard to contact (K), and because process-oriented information is usually scarce in academic or grey literature. To redress this gap, we believe that such data should be required in the Checklist for One Health Epidemiological Reporting of Evidence (COHERE) guidelines (10).

## Conclusion

We conclude that systems thinking and associated practices for OH require not only specific education in OH core competencies, but also methodological and institutional measures to endorse broad participation (3, 5, 22, 30). Particular attention should be given to conceiving initiatives as cyclic iterative processes and including evaluation as a collective learning opportunity. In this spirit, reporting knowledge integration processes should be included in the COHERE guidelines (10).

The two tools employed in this study were successful in triggering reflexive dialogues on the facilitation of knowledge integration, and in enabling co-production of improvements. A key factor was the social, didactic, and emotional competence of the evaluator(s). A further challenge is the scalability and comparability of the results obtained with the tools. More work should be invested in refining the indicator scales and establishing benchmarks. Also, how the size of initiatives affects their ability to integrate knowledge remains to be investigated, since large-scale initiatives have obvious advantages to affect broad systems, identify and engage stakeholders sustainably and mobilise significant resources and competencies, while smaller initiatives favour personal contact which support common group learning, internal sharing, and deep reflection required for bridging processes (31).

## Data Availability Statement

The raw data supporting the conclusions of this article will be made available by the authors, without undue reservation.

## Author Contributions

MH, JB, and SR conceived the research and the manuscript. MH and SR analysed the data and drafted the manuscript. MH, JB, and JZ established contact with the evaluated research initiatives. NP and JZ coordinated field visits, seminars, facilitated in kind support, and established local contacts. MH coordinated the research and conducted the fieldwork. SD, LF, ML, KL, TM, CM, KM, RÖ, NP, and JZ discussed the methodology, contributed to fieldwork, and commented on the manuscript. All authors read and approved the final manuscript.

## Conflict of Interest

The authors declare that the research was conducted in the absence of any commercial or financial relationships that could be construed as a potential conflict of interest.

## Supplementary Material

The EVOLvINC questionnaire,

The Excel workbook with data aggregation and analysis, i.e., sheets

- EVOLvINC aggregation (Table 4).
- NEOH aggregation
- Criteria EVOLvINC group
- Indicators EVOLvINC group
