# Supplementary material for: System Thinking and Citizen Participation Is Still Missing in One Health Initiatives – Lessons From Fifteen Evaluations
Source: Front Public Health. 2021 Jun 4;9:653398. doi: 10.3389/fpubh.2021.653398 (PMC8211880; doi:10.3389/fpubh.2021.653398)
Supplement: Supplementary file 1 [file Data_Sheet_1.PDF]

Questionnaire for the formative evaluation of One Health Surveillance initiatives

The International Society for Disease Surveillance's One Health (OH) working group aims to support and strengthen initiatives in OH Surveillance. To this end, it is collaborating with the Network for the Evaluation of One Health (NEOH, further information: http://neoh.onehealthglobal.net/)). This collaboration is based on the insight that the capability to integrate knowledge across disciplines, sectors and institutions throughout the project cycle is a core challenge for One Health surveillance. Therefore, it aims at raising the awareness of this challenge, and to validate a novel approach for assessing and increasing the capacity of OH surveillance initiatives to integrate knowledge across disciplines, sectors and institutions. To learn how knowledge integration is implemented in your initiative, this questionnaire assesses six generic features that are characteristic of OH initiatives and which facilitate knowledge integration. Evaluating these core features through pertinent indicators will contribute to developing and disseminating quality standards and to assessing the added benefits of OH Surveillance initiatives.

Having presented you with initial documentation and input material on knowledge integration in One Health, this questionnaire aims at validating the used indicators and collects baseline data about your initiative. After you receive this questionnaire, an interview with an external evaluator from NEOH will serve to fill this questionnaire, and to address any pending uncertainties. A final and concluding discussion will serve to feed back our results on the capacity for knowledge integration in your OH surveillance initiative, and to discuss the overall approach with regard to the insights and potential implications that you might see in this process for conducting your initiative. Therefore, the overall process helps us to learn whether people implementing OH surveillance initiatives perceive this approach as beneficial.

We thank you in advance for completing this questionnaire, and for supporting ISDS's OH working group. This is an opportunity for you to learn about our approaches, and to get in closer contact with ISDS's OH working group. It also will allow you to reflect on your project, and to develop a novel perspective on potential strengths and weaknesses. We offer each participating initiative to name one researcher as a co-author for a potential scientific publication. Thus, your participation is also an opportunity to highlight your project in publications aimed at academic and public audiences. In case there is a need to treat data or results with confidentiality, please let us know in advance. If you have any questions or would like more information, please send an email to: ***martin.hitziger2(at)uzh.ch***.

For background and references on underlying perspectives and models, please see:
Rüegg SR *et al.* A blueprint to evaluate One Health. *Front. Public Heal.* 1–5 (2016)
Hitziger M *et al*. Towards the implementation of One Health by integrating knowledge in the entire policy cycle. WHO Bulletin (accepted).

1. Personal information and role of the respondent

1.1 Your name and title		1.2 Date, location
1.3 To which institution are you affiliated?	1.4 What is your current position?	1.5 Please state your email and phone contacts?
1.6 What is the title or name of the One Health Surveillance project or initiative that you are working in?		
1.7 Where does this initiative take place (country, district, city)?		
1.8 What is your role in this initiative?		
1.9 What would you consider your key contribution to this initiative?		

2. Definition of context and initiative

Suggested sources of information for section 2: Program leaders or program management, institutional vision/mission statements, written sources such as scientific articles and program reports, as appropriate. For conceptualizing responses to this section, please use a separate paper sheet / slide / blackboard to conceptualize a systems model in a causal lop diagram, if appropriate.

2.1 How do you define One Health Surveillance? This question refers to the conceptualization of One Health Surveillance on which your initiative is based.

2.2 What is the problem / information gap that your initiative addresses / tries to fill? This question refers to your conceptualization of the problem or information gap, which the initiative aims to alleviate or to improve.

2.3 Please describe the aetiology of the problem / disease in the specific context in which your initiative is embedded. Please describe any resources (stocks) or tangible and intangible components being required or produced during the processes that cause the problem / disease. In case the problem is an infectious disease, please make sure to consider all relevant human, animal and environmental reservoirs, transmission vectors and transmission pathways.

2.4 Please describe the processes that affect the problem / enhance or impede disease incidence and prevalence in the specific context in which your initiative is embedded. Please describe relevant medical, environmental, or other physical relationships as well as governance and decision making processes. In case the problem is an infectious disease, please make sure to consider factors that affect human, animal and environmental reservoirs, transmission vectors and transmission pathways.

2.5 Please describe relevant stakeholders and actors who affect, are affected by, or perceive themselves to be affected by this problem / disease and the relationships that influence it. Please focus on important societal groups, institutions or organizations. Please consider as ***stakeholders*** those individuals or institutions that affect or are affected by the problem that the initiative addresses. ***Actors*** are a subset of the stakeholders, who affect the problem that the initiative addresses. In contrast to ***team members***, they represent the interests and perspectives of societal groups and institutions rather than acting under the direction of the project or program managers of the initiative. In case the problem is an infectious disease, please make sure to consider stakeholders and actors who affect, or are affected by human, animal and environmental reservoirs, transmission vectors and transmission pathways.

2.6 What are the overall objectives of the initiative? This question refers to the immediate outputs and outcomes that your initiative aspires to achieve. In case the problem is an infectious disease, please describe how the initiative aims to increase the understanding of, or the responses to human, animal and environmental reservoirs, transmission vectors and transmission pathways.

2.7 How would achieving the initiatives' objectives lead to wider impacts? This question refers to the initiative's theory of change, i.e. the mechanisms by which the initiatives' outputs and outcomes translate into wider impacts beyond the initiatives' control to resolve the shortcoming or gaps that it addresses. In case the problem is a disease, please describe how the initiative would lead to reducing its prevalence and incidence.

2.8 How do you measure the success of your initiative? This question refers to the initiative's criteria of success – how you judge that you have achieved your objectives. In case the problem is an infectious disease, please describe how you assess the initiatives' ability to identify risks and to improve responses to human, animal and environmental reservoirs, transmission vectors and transmission pathways.

2.9 What do you consider the most important benefits / accomplishments of this initiative? This question refers to accomplishments of the initiatives' process, outcomes and impacts up to date. It serves to complement or contrast with the envisioned / intended objectives. It refers both to process, expected outcomes as measured by the criteria of success, impacts as specified in the theory of change, and unintended or unexpected consequences. Please provide only observed benefits. Please do not provide planned benefits that are not completed or have not been operational long enough to provide observed benefits.

2.10 What do you consider the most important challenges / shortcomings of this initiative? This question refers to shortcomings of the initiatives' process, outcomes and impacts up to date. It serves to complement or contrast with the envisioned / intended objectives. It refers both to shortcomings of process, expected outcomes as measured by the criteria of success, impacts as specified in the theory of change, and unintended or unexpected consequences. Please provide only observed benefits. Please do not provide planned benefits that are not completed or have not been operational long enough to provide observed benefits.

3. Thinking

INTEGRATED APPROACH TO HEALTH, ENVIRONMENT AND SUSTAINABILITY

Suggested sources of information: Program leaders or program management, institutional vision/mission/platform/activities, written sources.

3.1 **Which aspects of One Health and the three pillars of sustainability does the problem affect?** Please tick as many as appropriate. The three pillars of sustainability are included here, since some One Health initiatives address societal or economic objectives.

One Health:

- ☐ human health
- ☐ animal health
- ☐ environmental health

- ☐ don't know / NA

Sustainability:

- ☐ environment
- ☐ society
- ☐ economy

- ☐ don't know / NA

3.2 **Does the initiative comprehensively translate the problem into scientific or developmental questions?** This question asks, whether the research questions and developmental questions reflect the complexity of different aspects of the problem.

- ☐ scientific or developmental questions do **not** address **any** aspects the problem
- ☐ scientific or developmental questions address **few** aspects of the problem
- ☐ scientific or developmental questions address **many** aspects of the problem
- ☐ scientific or developmental questions address **all** aspects of the problem

- ☐ don't know / NA

3.3 **Does the initiative bring innovation on the state of knowledge or tackling the problem?** Innovation can refer to either objectives, institutional platform, data, or methods. Lacking generalized scales for "innovation", this question relies on expert judgement of the evaluator, and can only be answered by comparing different projects.

☐ no innovation ☐ some innovation ☐ substantial innovation ☐ ground breaking ☐ don't know / NA

BROADNESS OF THE INITIATIVE AND MATCH TO CONTEXT

Suggested sources of information: Program leaders or program management, summaries of project design and set-up, written sources describing the context in which the initiative operates. If possible, the scores should be validated with stakeholders and actors who have insight in relevant policy / societal decision making processes.

3.4 Please use **column 1 to 4 of Table 1 (next pages)** to specify how the initiative's **OBJECTIVES** aim to address different **DIMENSIONS** of the problem, and which **SCALES** are suitable to measure its impact (there can be more than one scale per dimension and more than one dimension per objective). A "dimension" groups entities that can be captured by the same scale or concept. It is up to the respondent to introduce dimensions that express the project's objectives. The concept of scale refers to the combination of the level of analysis and the level of observation. Start by specifying objectives and by selecting which dimensions are considered by the initiative. Describe the relevant dimensions for the initiative (there may be fewer or more than the lines in table 1). Consider the theory of change. What is the initiative aiming to have an impact on? And which dimensions may support or limit the outcomes and impacts of the initiative?

Examples of dimensions could be: geographical space (scales within this dimension could be e.g. local, regional, global….), time (scales e.g. hours, days, years, centuries…), dimension of life (scales: e.g. cells tissues, organs, individuals, populations, ecosystems….), network/organisation (e.g. network of researchers, individuals from the public, institutions and national ministries, laboratories...), economy (e.g. farm level, sector-wise, national, regional, international trade, benefits, costs....), legislation (e.g. bilateral agreements, national laws), governance (political dimension, e.g. international/national strategy, local research project, specific task/work package), value constructs (e.g. interest groups, NGOs, religious beliefs, international standards......), collaborative capabilities (trust, networks, mutual understanding,…), or any other.

Please use **column 4** to list the **ACTORS** and **STAKEHOLDERS** (individuals, groups, or institutions that affect, or are affected by the system that the initiative targets), ***that are involved in the initiative and relevant for achieving each objective***. Please consider representatives of different academic disciplines, societal sectors, governmental or corporate organizations, for-profit or non-profit institutions and interest groups, as well as the general public as potential stakeholders.

3.5 Please use **column 5 of Table 1 (next pages)** to score the importance of the initiative's objectives by ticking the boxes on a scale from "no relevance" to "essential".

3.6 Please use **column 6 of Table 1 (next pages)** to score how strongly it would affect the outcomes of the initiative, if any particular objective was excluded? Please score on a scale from "no relevance" to "essential".

3.7 Please use **column 7 of Table 1 (next pages)** to score how strongly the initiative is affected by each dimension? Please score on a scale from "no relevance" to "essential".

3.8 Please use **column 8 of Table 1 (next pages)** to score how well the importance of each dimension matches the degree that the initiative affects or is affected by the dimension? This question aims at scoring the match between the initiative and the system within which it operates. Please consider the scores from questions 3.5 to 3.7 to assess the match and score on a scale from "no match" to "perfect match".

TABLE 1 (example sheet)

1) Objective (Q. 3.4)	*2) Dimension (Q. 3.4)*	*3) Scales (Q. 3.4)*	*4) Involved stakeholder (Q. 3.4)*	*6) Importance (Q. 3.5)*	*7) Effect of excluding objective (Q. 3.6)*	*8) Effect of dimension on initiative (Q. 3.7)*	*9) Initiative-context match (Q. 3.8)*	*10) Planning supports objective (Q. 4.10)*	*11) Stakeholder involvement (Q. 5.5)*
Reduce prevalence of pathogen in vector species	Animal Health	Pathogen prevalence at various spatial scales (communities, villages, towns, regions surrounding them, ecosystems)	Community members, farmers	☐ no relevance<br>☐ some<br>☐ highly<br>☐ essential<br><br>☐ don't know	☐ no relevance<br>☐ some<br>☐ highly<br>☐ essential<br><br>☐ don't know	☐ no relevance<br>☐ some<br>☐ highly<br>☐ essential<br><br>☐ don't know	☐ no match<br>☐ weak<br>☐ strong<br>☐ perfect<br><br>☐ don't know	☐ not supportive<br>☐ somewhat<br>☐ strongly supp.<br>☐ fully supp.<br><br>☐ don't know	☐ information<br>☐ consultation<br>☐ collaboration<br>☐ empowerment<br><br>☐ don't know
Reduce abundance of vector species	Environmental Management	Vector abundance at various spatial scales (communities, villages, towns, regions surrounding them, ecosystems)	Community members, farmers, vector control agencies (local NGO's, environmental management agencies)	☐ no relevance<br>☐ some<br>☐ highly<br>☐ essential<br><br>☐ don't know	☐ no relevance<br>☐ some<br>☐ highly<br>☐ essential<br><br>☐ don't know	☐ no relevance<br>☐ some<br>☐ highly<br>☐ essential<br><br>☐ don't know	☐ no match<br>☐ weak<br>☐ strong<br>☐ perfect<br><br>☐ don't know	☐ not supportive<br>☐ somewhat<br>☐ strongly supp.<br>☐ fully supp.<br><br>☐ don't know	☐ information<br>☐ consultation<br>☐ collaboration<br>☐ empowerment<br><br>☐ don't know
Reduce incidence in human reservoir hosts	Human Health<br><br>Local Medical Capacity	Disease incidence in people in local communities, towns, regions<br><br>Local equipment and skills for appropriate medial responses	Local and regional public health agencies, local health care providers, community members	☐ no relevance<br>☐ some<br>☐ highly<br>☐ essential<br><br>☐ don't know	☐ no relevance<br>☐ some<br>☐ highly<br>☐ essential<br><br>☐ don't know	☐ no relevance<br>☐ some<br>☐ highly<br>☐ essential<br><br>☐ don't know	☐ no match<br>☐ weak<br>☐ strong<br>☐ perfect<br><br>☐ don't know	☐ not supportive<br>☐ somewhat<br>☐ strongly supp.<br>☐ fully supp.<br><br>☐ don't know	☐ information<br>☐ consultation<br>☐ collaboration<br>☐ empowerment<br><br>☐ don't know
Improve local awareness and induce behavioural change	Sociocultural<br><br>Behavioural	Dissemination efforts to enhance public understanding of disease<br><br>Observed behavioural change	Local population, public health authorities, school masters, public at large	☐ no relevance<br>☐ some<br>☐ highly<br>☐ essential<br><br>☐ don't know	☐ no relevance<br>☐ some<br>☐ highly<br>☐ essential<br><br>☐ don't know	☐ no relevance<br>☐ some<br>☐ highly<br>☐ essential<br><br>☐ don't know	☐ no match<br>☐ weak<br>☐ strong<br>☐ perfect<br><br>☐ don't know	☐ not supportive<br>☐ somewhat<br>☐ strongly supp.<br>☐ fully supp.<br><br>☐ don't know	☐ information<br>☐ consultation<br>☐ collaboration<br>☐ empowerment<br><br>☐ don't know
Institutionalizing surveillance mechanism	Institution building<br><br>Governance<br><br>Economics<br><br>Data management	Speed of information flow<br><br>Efficiency of interagency monitoring efforts<br><br>Surveillance data<br><br>Database integration	Local surveillance & control agencies, WHO experts, national public health administration	☐ no relevance<br>☐ some<br>☐ highly<br>☐ essential<br><br>☐ don't know	☐ no relevance<br>☐ some<br>☐ highly<br>☐ essential<br><br>☐ don't know	☐ no relevance<br>☐ some<br>☐ highly<br>☐ essential<br><br>☐ don't know	☐ no match<br>☐ weak<br>☐ strong<br>☐ perfect<br><br>☐ don't know	☐ not supportive<br>☐ somewhat<br>☐ strongly supp.<br>☐ fully supp.<br><br>☐ don't know	☐ information<br>☐ consultation<br>☐ collaboration<br>☐ empowerment<br><br>☐ don't know

TABLE 1

1) Objective (Q. 3.4)	***2) Dimension (Q. 3.4)***	***3) Scales (Q. 3.4)***	***4) Involved stakeholder (Q. 3.4)***	***6) Importance (Q. 3.5)***	***7) Effect of excluding objective (Q. 3.6)***	***8) Effect of dimension on initiative (Q. 3.7)***	***9) Initiative-context match (Q. 3.8)***	***10) Planning supports objective (Q. 4.10)***	***11) Stakeholder involvement (Q. 5.5)***
				☐ no relevance<br>☐ some<br>☐ highly<br>☐ essential<br><br>☐ don't know	☐ no relevance<br>☐ some<br>☐ highly<br>☐ essential<br><br>☐ don't know	☐ no relevance<br>☐ some<br>☐ highly<br>☐ essential<br><br>☐ don't know	☐ no match<br>☐ weak<br>☐ strong<br>☐ perfect<br><br>☐ don't know	☐ not supportive<br>☐ somewhat<br>☐ strongly supp.<br>☐ fully supp.<br><br>☐ don't know	☐ information<br>☐ consultation<br>☐ collaboration<br>☐ empowerment<br><br>☐ don't know
				☐ no relevance<br>☐ some<br>☐ highly<br>☐ essential<br><br>☐ don't know	☐ no relevance<br>☐ some<br>☐ highly<br>☐ essential<br><br>☐ don't know	☐ no relevance<br>☐ some<br>☐ highly<br>☐ essential<br><br>☐ don't know	☐ no match<br>☐ weak<br>☐ strong<br>☐ perfect<br><br>☐ don't know	☐ not supportive<br>☐ somewhat<br>☐ strongly supp.<br>☐ fully supp.<br><br>☐ don't know	☐ information<br>☐ consultation<br>☐ collaboration<br>☐ empowerment<br><br>☐ don't know
				☐ no relevance<br>☐ some<br>☐ highly<br>☐ essential<br><br>☐ don't know	☐ no relevance<br>☐ some<br>☐ highly<br>☐ essential<br><br>☐ don't know	☐ no relevance<br>☐ some<br>☐ highly<br>☐ essential<br><br>☐ don't know	☐ no match<br>☐ weak<br>☐ strong<br>☐ perfect<br><br>☐ don't know	☐ not supportive<br>☐ somewhat<br>☐ strongly supp.<br>☐ fully supp.<br><br>☐ don't know	☐ information<br>☐ consultation<br>☐ collaboration<br>☐ empowerment<br><br>☐ don't know
				☐ no relevance<br>☐ some<br>☐ highly<br>☐ essential<br><br>☐ don't know	☐ no relevance<br>☐ some<br>☐ highly<br>☐ essential<br><br>☐ don't know	☐ no relevance<br>☐ some<br>☐ highly<br>☐ essential<br><br>☐ don't know	☐ no match<br>☐ weak<br>☐ strong<br>☐ perfect<br><br>☐ don't know	☐ not supportive<br>☐ somewhat<br>☐ strongly supp.<br>☐ fully supp.<br><br>☐ don't know	☐ information<br>☐ consultation<br>☐ collaboration<br>☐ empowerment<br><br>☐ don't know
				☐ no relevance<br>☐ some<br>☐ highly<br>☐ essential<br><br>☐ don't know	☐ no relevance<br>☐ some<br>☐ highly<br>☐ essential<br><br>☐ don't know	☐ no relevance<br>☐ some<br>☐ highly<br>☐ essential<br><br>☐ don't know	☐ no match<br>☐ weak<br>☐ strong<br>☐ perfect<br><br>☐ don't know	☐ not supportive<br>☐ somewhat<br>☐ strongly supp.<br>☐ fully supp.<br><br>☐ don't know	☐ information<br>☐ consultation<br>☐ collaboration<br>☐ empowerment<br><br>☐ don't know

TABLE 1 (CONTINUED)

1) Objective (Q. 3.4)	*2) Dimension (Q. 3.4)*	*3) Scales (Q. 3.4)*	*4) Involved stakeholder (Q. 3.4)*	*6) Importance (Q. 3.5)*	*7) Effect of excluding objective (Q. 3.6)*	*8) Effect of dimension on initiative (Q. 3.7)*	*9) Initiative-context match (Q. 3.8)*	*10) Planning supports objective (Q. 4.10)*	*11) Stakeholder involvement (Q. 5.5)*
				☐ no relevance<br>☐ some<br>☐ highly<br>☐ essential<br><br>☐ don’t know	☐ no relevance<br>☐ some<br>☐ highly<br>☐ essential<br><br>☐ don’t know	☐ no relevance<br>☐ some<br>☐ highly<br>☐ essential<br><br>☐ don’t know	☐ no match<br>☐ weak<br>☐ strong<br>☐ perfect<br><br>☐ don’t know	☐ not supportive<br>☐ somewhat<br>☐ strongly supp.<br>☐ fully supp.<br><br>☐ don’t know	☐ information<br>☐ consultation<br>☐ collaboration<br>☐ empowerment<br><br>☐ don’t know
				☐ no relevance<br>☐ some<br>☐ highly<br>☐ essential<br><br>☐ don’t know	☐ no relevance<br>☐ some<br>☐ highly<br>☐ essential<br><br>☐ don’t know	☐ no relevance<br>☐ some<br>☐ highly<br>☐ essential<br><br>☐ don’t know	☐ no match<br>☐ weak<br>☐ strong<br>☐ perfect<br><br>☐ don’t know	☐ not supportive<br>☐ somewhat<br>☐ strongly supp.<br>☐ fully supp.<br><br>☐ don’t know	☐ information<br>☐ consultation<br>☐ collaboration<br>☐ empowerment<br><br>☐ don’t know
				☐ no relevance<br>☐ some<br>☐ highly<br>☐ essential<br><br>☐ don’t know	☐ no relevance<br>☐ some<br>☐ highly<br>☐ essential<br><br>☐ don’t know	☐ no relevance<br>☐ some<br>☐ highly<br>☐ essential<br><br>☐ don’t know	☐ no match<br>☐ weak<br>☐ strong<br>☐ perfect<br><br>☐ don’t know	☐ not supportive<br>☐ somewhat<br>☐ strongly supp.<br>☐ fully supp.<br><br>☐ don’t know	☐ information<br>☐ consultation<br>☐ collaboration<br>☐ empowerment<br><br>☐ don’t know
				☐ no relevance<br>☐ some<br>☐ highly<br>☐ essential<br><br>☐ don’t know	☐ no relevance<br>☐ some<br>☐ highly<br>☐ essential<br><br>☐ don’t know	☐ no relevance<br>☐ some<br>☐ highly<br>☐ essential<br><br>☐ don’t know	☐ no match<br>☐ weak<br>☐ strong<br>☐ perfect<br><br>☐ don’t know	☐ not supportive<br>☐ somewhat<br>☐ strongly supp.<br>☐ fully supp.<br><br>☐ don’t know	☐ information<br>☐ consultation<br>☐ collaboration<br>☐ empowerment<br><br>☐ don’t know
				☐ no relevance<br>☐ some<br>☐ highly<br>☐ essential<br><br>☐ don’t know	☐ no relevance<br>☐ some<br>☐ highly<br>☐ essential<br><br>☐ don’t know	☐ no relevance<br>☐ some<br>☐ highly<br>☐ essential<br><br>☐ don’t know	☐ no match<br>☐ weak<br>☐ strong<br>☐ perfect<br><br>☐ don’t know	☐ not supportive<br>☐ somewhat<br>☐ strongly supp.<br>☐ fully supp.<br><br>☐ don’t know	☐ information<br>☐ consultation<br>☐ collaboration<br>☐ empowerment<br><br>☐ don’t know

TABLE 1 (CONTINUED)

1) Objective (Q. 3.4)	*2) Dimension (Q. 3.4)*	*3) Scales (Q. 3.4)*	*4) Involved stakeholder (Q. 3.4)*	*6) Importance (Q. 3.5)*	*7) Effect of excluding objective (Q. 3.6)*	*8) Effect of dimension on initiative (Q. 3.7)*	*9) Initiative-context match (Q. 3.8)*	*10) Planning supports objective (Q. 4.10)*	*11) Stakeholder involvement (Q. 5.5)*
				☐ no relevance<br>☐ some<br>☐ highly<br>☐ essential<br><br>☐ don't know	☐ no relevance<br>☐ some<br>☐ highly<br>☐ essential<br><br>☐ don't know	☐ no relevance<br>☐ some<br>☐ highly<br>☐ essential<br><br>☐ don't know	☐ no match<br>☐ weak<br>☐ strong<br>☐ perfect<br><br>☐ don't know	☐ not supportive<br>☐ somewhat<br>☐ strongly supp.<br>☐ fully supp.<br><br>☐ don't know	☐ information<br>☐ consultation<br>☐ collaboration<br>☐ empowerment<br><br>☐ don't know
				☐ no relevance<br>☐ some<br>☐ highly<br>☐ essential<br><br>☐ don't know	☐ no relevance<br>☐ some<br>☐ highly<br>☐ essential<br><br>☐ don't know	☐ no relevance<br>☐ some<br>☐ highly<br>☐ essential<br><br>☐ don't know	☐ no match<br>☐ weak<br>☐ strong<br>☐ perfect<br><br>☐ don't know	☐ not supportive<br>☐ somewhat<br>☐ strongly supp.<br>☐ fully supp.<br><br>☐ don't know	☐ information<br>☐ consultation<br>☐ collaboration<br>☐ empowerment<br><br>☐ don't know
				☐ no relevance<br>☐ some<br>☐ highly<br>☐ essential<br><br>☐ don't know	☐ no relevance<br>☐ some<br>☐ highly<br>☐ essential<br><br>☐ don't know	☐ no relevance<br>☐ some<br>☐ highly<br>☐ essential<br><br>☐ don't know	☐ no match<br>☐ weak<br>☐ strong<br>☐ perfect<br><br>☐ don't know	☐ not supportive<br>☐ somewhat<br>☐ strongly supp.<br>☐ fully supp.<br><br>☐ don't know	☐ information<br>☐ consultation<br>☐ collaboration<br>☐ empowerment<br><br>☐ don't know
				☐ no relevance<br>☐ some<br>☐ highly<br>☐ essential<br><br>☐ don't know	☐ no relevance<br>☐ some<br>☐ highly<br>☐ essential<br><br>☐ don't know	☐ no relevance<br>☐ some<br>☐ highly<br>☐ essential<br><br>☐ don't know	☐ no match<br>☐ weak<br>☐ strong<br>☐ perfect<br><br>☐ don't know	☐ not supportive<br>☐ somewhat<br>☐ strongly supp.<br>☐ fully supp.<br><br>☐ don't know	☐ information<br>☐ consultation<br>☐ collaboration<br>☐ empowerment<br><br>☐ don't know
				☐ no relevance<br>☐ some<br>☐ highly<br>☐ essential<br><br>☐ don't know	☐ no relevance<br>☐ some<br>☐ highly<br>☐ essential<br><br>☐ don't know	☐ no relevance<br>☐ some<br>☐ highly<br>☐ essential<br><br>☐ don't know	☐ no match<br>☐ weak<br>☐ strong<br>☐ perfect<br><br>☐ don't know	☐ not supportive<br>☐ somewhat<br>☐ strongly supp.<br>☐ fully supp.<br><br>☐ don't know	☐ information<br>☐ consultation<br>☐ collaboration<br>☐ empowerment<br><br>☐ don't know

PROJECT DESIGN

Suggested sources of information: Program leaders or program management, summaries of project design and set-up, written sources. If possible, the score should be validated with stakeholders and team members that have insight in relevant methods, and societal decision processes.

3.9 **How are objectives and their relative importance established?** Conflicting objectives, limited resources or external constraints require implicit or explicit trade-offs that reflect the importance of objectives. Please score the attention that was/is devoted to developing and understanding objectives, trade-offs and degrees of importance.

☐ no attention ☐ implicit attention without explicit consideration ☐ explicit consideration without formal analysis ☐ formal analysis ☐ don't know / NA

3.10 **Has a theory of change been elaborated to match the objectives of the initiative?** A theory of change specifies how the initiative will produce impact that transforms the problem that it addresses. Please score the attention that was devoted to understanding and validating the changes that the initiative needs to bring about to achieve its objectives.

☐ no attention ☐ implicit attention without explicit consideration ☐ explicit consideration without formal analysis ☐ formal analysis ☐ don't know / NA

3.11 **How do the objectives and the theory of change reflect multiple perspectives, value systems and beliefs?** This question refers to the openness and inclusiveness of the process of project set-up.

☐ they reflect the perspective of the project management
☐ they were decided by the project management but without dialogue or negotiation with stakeholders
☐ they were decided by the project management after dialogue with stakeholders, but without negotiation
☐ they were negotiated and agreed upon by project management and stakeholders

☐ don't know / NA

3.12 How do the methods, scales and criteria of success reflect multiple perspectives, value systems and beliefs? This question refers to the openness and inclusiveness of designing the project implementation phase.

☐ they reflect the perspective of the project management
☐ they were decided by the project management but without dialogue or negotiation with stakeholders
☐ they were decided by the project management after dialogue with stakeholders, but without negotiation
☐ they were negotiated and agreed upon by project management and stakeholders

☐ don't know / NA

CONSIDERATION OF SYSTEM FEATURES

Suggested sources of information: Program leaders or program management, summaries of project design and set-up, written sources describing the context in which the initiative operates. If possible, the score should be validated with stakeholders that have insight in relevant policy / societal decision making processes.

3.13 Is the problem that the initiative addresses an event, a pattern, or a structure? ***Events*** are singular; and we can only react to events. ***Patterns*** let us understand reality at a deeper level, i.e. trends, which are changes over time to which we can adapt. Thinking at a ***structural*** level means thinking in terms of causal connections. They are the key to lasting, high-leverage change on how the system operates.

☐ events ☐ patterns ☐ structures ☐ don't know / NA

3.14 **How are time delays between different processes in the system considered?** Please score the attention that was devoted to understanding time scales and delays between different processes.

☐ no attention ☐ implicit attention without explicit consideration ☐ explicit consideration without formal analysis ☐ formal analysis ☐ don't know / NA

3.15 **How are feedback loops and causal interactions in the system considered?** Feedback loops are interactions between two or more factors that mutually reinforce (positive feedback loop), or control (negative feedback loop) each other. Please score the attention that was devoted to understanding feedback loops and causal interactions.

☐ no attention ☐ implicit attention without explicit consideration ☐ explicit consideration without formal analysis ☐ formal analysis ☐ don't know / NA

3.16 **Where is the initiative situated in relation to the chain of events causing the problem?** This question should be answered by putting into perspective the theory of change, the project impacts according to its objectives, and the problem that has been identified in the system.

☐ correcting damage ☐ preventing damage ☐ redirecting the problem ☐ removing the cause ☐ don't know / NA

4. Planning

IDENTIFICATION AND ENGAGEMENT OF SECTORS, ACTORS AND STAKEHOLDERS

Suggested sources of information: Program leaders or program management. Contracts MoU's or other forms of written agreements and commitments, if available. If possible, the score should be validated with stakeholders that have insight in relevant policy / societal decision making processes.

4.1 **How are sectors and disciplines identified, that affect or are affected by the problem that the initiative targets and are thus relevant for achieving its objectives and for leveraging impact?** Sector refer to societal or institutional groups that share common aims or similar institutional structures, such as banking, industry, or public governance authorities. Disciplines refer to scientific specializations such as medicine (and veterinary medicine), mathematics, ecology, chemistry, environmental science.

☐ no identification of relevant sectors and disciplines
☐ ad hoc identification of some sectors and disciplines by project management
☐ informal process by project management, aimed at comprehensive selection of relevant sectors and disciplines
☐ formal analysis, consultations with external experts/advisors or participatory process

☐ don't know / NA

4.2 **How are stakeholders and actors identified?** These are individuals, groups or institutions, who have a vested interest, or dispose of knowledge which is relevant for achieving the initiative's objectives and for leveraging impact.

☐ no stakeholder identification
☐ ad hoc identification of some stakeholders by project management
☐ informal process by project management aimed at comprehensive selection of relevant affected stakeholders
☐ formal analysis, consultations with external experts/advisors or participatory process

☐ don't know / NA

4.3 How is stakeholder commitment assured? This question refers to the degree of formality and institutional commitment invested in / required from stakeholder groups.

- ☐ no stakeholder engagement
- ☐ ad hoc invitations according to immediate opportunity
- ☐ engagement according to informal agreements
- ☐ formal engagement negotiated with stakeholders and institutional commitments to ensure accountability

- ☐ don't know / NA

REFLEXIVITY AND ADAPTIVENESS

Suggested sources of information: Program leaders or program management. Written initiative proposals or applications, if available. If possible, the score should be validated with stakeholders and team members, as appropriate.

4.4 Which opportunities for reflection and self-assessment does the initiative provide? This question refers to the effort and opportunities within the initiative to enable reflexivity among team members and stakeholders. It can refer to both formal arrangements or other occasions, in which project management, team members and stakeholders of the initiative ask for, and receive feedback on process, accomplishments and future directions.

- ☐ no opportunities for self-assessment and reflection
- ☐ informal ad hoc opportunities for internal dialogue, feedback and reflection
- ☐ structured process for internal dialogue, feedback and reflection
- ☐ structured process for internal dialogue and feedback that requires team members and stakeholders to personally experience and take perspectives that are different from one's own (e.g. temporary role changes, joint field work etc.)

- ☐ don't know / NA

4.5 How flexible is the project execution and timeline to respond to internal or external changes in the short-, mid-, and long term? This question refers to opportunities within the initiative to adapt to changing external (e.g. environmental or epidemiological health situation) or internal conditions (e.g. policy, funding, or management structures). It can both refer to institutional arrangements for future adaptations, or past experience.

	**short-term** <= 1 year	**mid-term** 1-3 years	**long-term** >= 3 years
no possibility to adapt the original plan during its execution	☐	☐	☐
minor adjustments possible according to circumstances	☐	☐	☐
considerable possibility to adjust according to circumstances	☐	☐	☐
formal iterative decision making and management process to regularly reconsider and adapt project execution	☐	☐	☐
don’t know / NA	☐	☐	☐

COMPETENCES

Suggested sources of information: written sources on team structure, skills, manuals, methods guidelines and project objectives if available. A broad selection of interviewed team members from different sectors and hierarchical levels. Structured tools such as required competences per objective tables may be used as appropriate.

4.6 How adequate are the competences of team members and actors to achieve the objectives? There is no generalizable scale for the adequacy of competences across different contexts, and this question can easily lead to strategically biased responses. Therefore, the scale is up to the evaluator’s professional judgement, which shall take into account local context and perspectives, and draw on a broad data basis.

☐ inadequate ☐ partly adequate ☐ mostly adequate ☐ entirely adequate ☐ don’t know / NA

4.7 **How adequate are the methods to achieve the objectives?** There is no generalizable scale for the adequacy of methods across different contexts, and this question can easily lead to strategically biased responses. Therefore, the scale is up to the evaluator's professional judgement, which shall take into account local context and perspectives, and draw on a broad data basis.

☐ inadequate ☐ partly adequate ☐ mostly adequate ☐ entirely adequate ☐ don't know / NA

RESOURCE ALLOCATION

Suggested sources of information: written sources source as budget proposals, timetables and duty schedules if available. A broad selection of interviewed team members from different sectors and hierarchical levels. Structured tools such as required budget per objective tables may be used as appropriate.

4.8 **How adequate are the budget allowances to achieve the objectives?** There is no generalizable scale for the adequacy of the allowances across different contexts, and this question can easily lead to strategically biased responses. Therefore, the scale is up to the evaluator's professional judgement, which shall take into account local context and perspectives, and draw on a broad data basis.

☐ inadequate ☐ partly adequate ☐ mostly adequate ☐ entirely adequate ☐ don't know / NA

4.9 **How adequate are the time allowances to achieve the objectives?** There is no generalizable scale for the adequacy of allowances across different contexts, and this question can easily lead to strategically biased responses. Therefore, the scale is up to the evaluator's professional judgement, which shall take into account local context and perspectives, and draw on a broad data basis.

☐ inadequate ☐ partly adequate ☐ mostly adequate ☐ entirely adequate ☐ don't know / NA

4.10 Going back to **TABLE 1**, please rate in **COLUMN 9** how far the planning was supportive to achieve each objective. Please consider (i) planned methods, (ii) available competencies, (iii) available time and (iv) budget allowances. Please orient your score for each objective at the weakest, least supportive of these four aspects. Lacking a generalized scale and requiring the aggregation of several considerations, this question requires particular professional judgement of the evaluator.

5. Organization

BRIDGING KNOWLEDGES

Suggested sources of information: project or program management, please validate with team members and stakeholders at different hierarchical levels.

5.1 **Which methods are used to 'bridge', 'link' or 'integrate' the knowledge of team members, actors and stakeholders?** Since terminology and methodology of knowledge integration are not commonly shared between different initiatives, considerable depth of inquiry and effort to translate interview responses into answers to this question are required from the evaluator. Answers to this question draw on the following sources:

- Scholz et al. 2002: Embedded case study methods: integrating quantitative and qualitative knowledge. Sage Publications.
- Bergmann et al. 2012: Methods for transdisciplinary research: a primer for practice. Frankfurt, New York: Campus.
- Hoffmann et al. 2017: Methods and procedures of transdisciplinary knowledge integration. Ecology and Society 22/1.

If appropriate, please tick multiple elements.

☐ none
☐ written information exchange (e.g. reports dissemination, social media networks, mail)
☐ face-to face networking & unstructured dialogue (e.g. receptions, information events)
☐ facilitated dialogue/moderation (e.g. workshops, focus groups, panel discussions)
☐ mediation through trusted bridge persons or boundary institutions
☐ joint definition of concepts and analytical frameworks
☐ joint formulation of objectives, research questions and hypotheses
☐ joint elaboration of theory of change, methods and assessment procedures
☐ joint conduction of fieldwork or joint elaboration of products (e.g. boundary objects, models, system dynamic graphs)
☐ changes of perspective / experiential encounters

☐ other, please specify:
☐ don't know / NA

5.2 **Which processes are used to 'bridge', 'link' or 'integrate' the knowledge of team members, actors and stakeholders?** Since terminology and methodology of knowledge integration are not commonly shared between different initiatives, considerable depth of inquiry and effort to translate interview responses into answers to this question are required from the evaluator. Answers to this question draw on the following sources:

- Rossini 1979: Frameworks for integrating interdisciplinary research. Research Policy 8/1.
- Hoffmann et al. 2017: Methods and procedures of transdisciplinary knowledge integration. Ecology and Society 22/1.

If appropriate, please tick multiple elements.

☐ none
☐ centralized integration through leader / project management
☐ integration through negotiation among experts
☐ common group learning

☐ other, please specify:
☐ don't know / NA

EXTERNAL STAKEHOLDER NETWORK

Suggested sources of information: project or program management, if possible validated with stakeholders.

5.3 **How frequently are stakeholders involved in the initiative?** This question refers to the frequency of any kind of interaction and information exchange between stakeholders and team members of the initiative, as they are elicited in the questions 5.1 and 5.2. In case the frequency differs between different stakeholders or stakeholder groups, please score the average frequency of interaction with each core stakeholder.

☐ never ☐ occasionally, but not on a regular basis ☐ in regular intervals but not frequently ☐ frequently ☐ don't know / NA

5.4 **How intense is the collaboration between different stakeholders in the initiative?** This question refers to the collaboration between stakeholders, rather than between the initiative and stakeholders. In case the intensity differs between different stakeholders or stakeholder groups, please score the average intensity of collaboration between core stakeholders.

☐ No contact or indirect information exchange through the initiative, but no face-to face participation
☐ Face-to face participation in events, but no joint task execution
☐ Joint task execution, but without an influence on decision making or project steering
☐ Joint steering, decision making, or co-leadership

☐ don't know / NA

5.5 **How intense is the involvement of stakeholders in the initiative?** Please go back to **TABLE 1**, and use **COLUMN 10** rate the stakeholder involvement for each objective. Please use the following scale: (i) no contact or stakeholders provide **information**, (ii) information flows in both directions (**consultation**), (iii) stakeholders contribute resources or task execution (**collaboration**), (iv) joint steering, decision making, or co-leadership (**empowerment**).

INTERNAL TEAM STRUCTURE

Please consider as team members those individuals, who participate in the initiative under the direction of the project or program management and contribute specific skills or fulfil specific duties to achieve the initiative's objectives. In contrast to stakeholders, they do not primarily participate in the initiative as representatives of interests and perspectives of societal groups or organizations. *Suggested sources of information: project or program management, please validate with members at different teams.*

5.6 **How many teams are present in the initiative?** Please consider as a team each sub-group of members of the initiative that are provided with resources and scope for decision making to achieve specific objectives, produce specific outputs, or to fulfil specific duties.

☐ zero ☐ one ☐ two-three ☐ more than three ☐ don't know / NA

5.7 **If more than one team are present, how are inter-team relations?** This question refers to the collaborative spirit between different teams. Since this question is prone to produce biased responses, it is therefore crucial to validate responses with various participants at different hierarchical levels. If relation between teams differ, please score an average value.

☐ competition ☐ ignorance ☐ mutual information ☐ mutual support ☐ don't know / NA

5.8 **How are the team objectives established?** This question explores the degree of formality employed in assigning team objectives.

☐ not at all ☐ implicitly assumed ☐ ad hoc agreed ☐ explicitly defined ☐ don't know / NA

5.9 **How are individual roles established and differentiated?** This question explores the degree of formality employed in assigning team member's roles and responsibilities.

☐ not at all ☐ implicitly assumed ☐ ad hoc agreed ☐ explicitly defined ☐ don't know / NA

5.10 **Are the teams recognized by stakeholders as clearly defined teams?** This question explores the degree of formality and distinctiveness that teams have within the initiative.

☐ not recognized ☐ recognized ☐ respected ☐ appreciated ☐ don't know / NA

6. Working

LEADERSHIP

Suggested sources of information: participants of the initiative from various specializations and hierarchical levels.

6.1 **Is the management structure appropriate to support the team and actors in achieving the initiative's objectives?** This question refers to the ability of the project management to coordinate and administer all initiative activities. Due to the lack of a generalizable scale to aggregate the various dimensions related to project coordination, this question relies on the evaluators judgement, and requires drawing on input from multiple initiative participants.

☐ inappropriate ☐ partially ☐ mostly ☐ fully appropriate ☐ don't know / NA

6.2 **How would you characterize the leadership approach to project management?** This question refers to definitions by Yukl (*Effective Leadership Behavior: What We Know and What Questions Need More Attention. Acad. of Manag. Perspect. 26/4: 66–85 (2012)*). It requires considerable inquiry on behalf of the evaluator. ***Task-oriented*** leadership focuses on accomplishing work in an efficient and reliable way. ***Relations-oriented*** leadership aims at increasing the quality of human resources and relations ("human capital"). ***Change-oriented*** leadership focuses on increased innovation, collective learning, and adaptation to the external environment. If appropriate, please tick multiple elements.

☐ no leadership ☐ task-oriented ☐ relationship-oriented ☐ change-oriented ☐ don't know / NA

6.3 **How open-minded is the leadership to creative input?** This question refers to the openness of the initiatives leadership to consider and test creative or unconventional ideas and input. To assess this question, it is suggested to ask participants for information and suggestions that were creative in content or did not follow conventional chains of command, and whether any of these led to surprising decisions, unconventional approaches differing from previous habits or protocols, or unexpected changes in project design, fieldwork and objectives.

☐ closed ☐ rarely open ☐ usually open ☐ frequently open-minded ☐ don't know / NA

6.4 **How flexible are internal hierarchies and decision making in adapting to circumstances and tasks?** The question refers to the degree of independence participants are given according to their expertise and skills, and the leaderships´ flexibility to devolve decision making according to need and context. To assess this question, it is suggested to ask for the degree of independence participants are given in specific realms, and for occasions, in which unconventional situations required deviating from chains of command in unforeseen ways.

☐ hierarchies are formalistic, top-down decision making
☐ hierarchies are formalistic, top-down decision making, but takes lower level's perspectives into account
☐ hierarchies are formalistic, but decisions reflect cross-hierarchical perspectives
☐ hierarchies are flexible and reflect the expertise and experience needed in specific situations

☐ don't know / NA

CONFLICT RESOLUTION

Suggested sources of information: Project or program management, please validate with participants from various specializations and hierarchical levels.

6.5 **How would you characterize the leadership approach to managing people?** There are different approaches to lead people towards achieving common objectives. 'No leadership' designates a project management that lacks successful strategies and approaches of exerting influence within the initiative. 'Controlling' leadership relies on order and control, 'rewarding' leadership relies on extrinsic (monetary or non-monetary) incentives. 'Inspirational' leadership relies on persuasion and creating enthusiasm and intrinsic commitment. If appropriate, please tick multiple elements.

☐ no leadership ☐ controlling ☐ rewarding ☐ inspiring ☐ don't know / NA

6.6 **How does the leadership manage tensions and conflicts?** This question addresses the degree to which project management involves conflicting partners to solve conflicts in the initiative. 'Evasion' designates a leadership that does not address tensions or conflicts within the initiative. 'Appeasement' is based on satisfying demands brought forward in a conflict without involving all parties. 'Imposition' prescribes certain solutions to conflicting parties. 'Mediation' negotiates solutions among conflicting parties. If appropriate, please tick multiple elements.

☐ evasion ☐ appeasement ☐ imposition ☐ mediation ☐ don't know / NA

6.7 **At what level are conflicts resolved?** This question addresses the degree of reflection and learning that project management derives from conflicts within the initiative. 'Hierarchical' refers to solutions that are prescribed without reflecting on sources and potential lessons. Reflection and learning can address mistakes and potential changes to 'factual' causes, can search for and address 'emotional' sources of conflict in individual personalities, or serve to build coherence, understanding, trust, and commitment across the entire team. If appropriate, please tick multiple elements.

☐ hierarchical ☐ factual ☐ emotional & personal growth ☐ team building ☐ don't know / NA

6.8 How does the team react to conflict? This question refers to conflict-related attitudes and group dynamics in the entire team of the initiative. Conflicts and sources of potential future conflicts such as unwelcome information can be seen as negative and to be avoided, thus leading to silencing or 'concealing'. 'Confrontation' spells conflict out, while focusing on the conflictual situation and a resistance to reflect own positions. 'Dialogue' spells conflict out, but with an attitude focused on solutions and reflecting own positions. Tolerant and resilient conflict behaviour addresses conflicts in dialogue, but also accepts differences in objectives and perceptions, acknowledges their justification and accommodates certain levels of ambiguity and diversity in interpreting conflictive situations. If appropriate, please tick multiple elements.

☐ concealing ☐ confrontation ☐ dialogue ☐ tolerance & resilience ☐ don't know / NA

POWER DISTRIBUTION

Suggested sources of information: data and written sources on decision making protocols and chains of command, participants of the initiative from various specializations and hierarchical levels.

6.9 How is the distribution of power or influence between team members and stakeholders from different… Please consider formal power (e.g. positions, hierarchies, budget responsibilities) as well as informal or "soft" forms of influence (e.g. openness to voice opinions, respect towards expertise and personality of team members, loyalties between team members).

**(i) disciplines?**	☐ balanced	☐ mostly balanced	☐ clustered	☐ concentrated	☐ don't know / NA
**(ii) sectors?**	☐ balanced	☐ mostly balanced	☐ clustered	☐ concentrated	☐ don't know / NA
**(iii) ethnicities?**	☐ balanced	☐ mostly balanced	☐ clustered	☐ concentrated	☐ don't know / NA
**(iv) social classes?**	☐ balanced	☐ mostly balanced	☐ clustered	☐ concentrated	☐ don't know / NA
**(v) genders?**	☐ balanced	☐ mostly balanced	☐ clustered	☐ concentrated	☐ don't know / NA
**(vi) cultures?**	☐ balanced	☐ mostly balanced	☐ clustered	☐ concentrated	☐ don't know / NA
**(vii) religions?**	☐ balanced	☐ mostly balanced	☐ clustered	☐ concentrated	☐ don't know / NA

7. Sharing

GENERAL INFORMATION

Suggested sources of information: formal documents or agreements between the program management, team members of the initiative and stakeholders, program management, please validate with team members and stakeholders.

7.1 How are agreements concerning information sharing established?

☐ not at all ☐ implicit agreement, without explicit negotiation ☐ explicit negotiation, but without a binding document ☐ binding document ☐ don't know / NA

7.2 Does the initiative have internal mechanisms to facilitate exchange of information within the initiative and are these used? E.g. newsletters, workshops, reports, publications, online sharing platform. Please only consider mechanisms which are also used.

☐ none ☐ few and irregular ☐ several and frequent ☐ continuous exchange ☐ don't know / NA

7.3 Does the initiative have external mechanisms to facilitate exchange of information beyond the initiative and are these used? E.g. newsletters, workshops, reports, publications, online sharing platform. Please only consider mechanisms which are also used.

☐ none ☐ few and irregular ☐ several and frequent ☐ continuous exchange ☐ don't know / NA

7.4 Are resources allocated to ensure information sharing? This refers to both monetary and non-monetary resources required for enabling information sharing.

☐ none ☐ insufficient ☐ sufficient ☐ plenty ☐ don't know / NA

DATA

Suggested sources of information: formal documents (if available), program management, please validate with team members and stakeholders (if appropriate).

7.5 Are procedures in place to ensure the quality of shared data? E.g. completeness, error-checking, clear and accurate descriptions of variables and of calculations, available documentation.

☐ none ☐ little ☐ considerable ☐ thorough ☐ don't know / NA

7.6 Are procedures in place to ensure safe and appropriate data storage and accessibility? E.g. is the storage safe and protected, is extraction of data feasible without access to data managers, or are expert managers readily available for extraction of data, is the process of data extraction bureaucratic/cumbersome/overly time-consuming.

☐ none ☐ little ☐ considerable ☐ thorough ☐ don't know / NA

7.7 How well / how much are data being shared within the initiative?

☐ not at all ☐ between few people ☐ between few groups ☐ within entire initiative ☐ don't know / NA

METHODS AND RESULTS

Suggested sources of information: program management, please validate with team members and stakeholders (if appropriate).

7.8 How well / how much are methods shared between people within the initiative?

☐ not at all ☐ between few people ☐ between few groups ☐ within entire initiative ☐ don't know / NA

7.9 How well / how much are results shared between people within the initiative?

☐ not at all ☐ between few people ☐ between few groups ☐ within entire initiative ☐ don't know / NA

INSTITUTIONAL MEMORY

Suggested sources of information: written documents (if available), program management, please validate with team members and stakeholders.

7.10 Does the initiative create or use long-term institutional knowledge reservoirs for data, methods and results? E.g. publications, detailed reports/manuals, database descriptions, standard operating procedures, introductions to inform new staff about essential procedures.

☐ not at all ☐ few ☐ several ☐ comprehensively ☐ don't know / NA

7.11 **Are procedures in place to safe-guard access to data, information and results in case of system change**? E.g. change of IT-system, data ownership, institutional organization.

☐ not at all ☐ few ☐ several ☐ comprehensively ☐ don't know / NA

8. Learning

INDIVIDUAL LEARNING

Suggested sources of information: program management, team members and stakeholders (if appropriate).

8.1 **How often do individuals receive information which may be understood and may potentially lead to learning, but it is not put into practice in or outside the initiative by the individuals (basic learning)?**

☐ never ☐ rarely ☐ regularly ☐ frequently ☐ don't know / NA

8.2 **How often is information understood, learnt and applied to improve procedures, competencies, technologies and paradigms without challenging the individuals' underlying beliefs and assumptions (adaptive learning)?**

☐ never ☐ rarely ☐ regularly ☐ frequently ☐ don't know / NA

8.3 **How often is information understood and learnt by individuals and applied to improve procedures, competencies, technologies and paradigms as a result of modified underlying beliefs and norms of individuals (generative learning)?**

☐ never ☐ rarely ☐ regularly ☐ frequently ☐ don't know / NA

TEAM LEARNING

Suggested sources of information: program management, please validate with team members and stakeholders (if appropriate).

8.4 How often do teams meet to exchange information for reporting purposes (basic learning)?

☐ never ☐ rarely ☐ regularly ☐ frequently ☐ don't know / NA

8.5 When teams meet, how often are different views presented, defended and discussed to find the best view to support decision making (adaptive learning)?

☐ never ☐ rarely ☐ regularly ☐ frequently ☐ don't know / NA

8.6 When teams meet, how often are complex issues explored through dissection of views and assumptions of team members resulting in a move towards building new ideas, views or approaches (generative learning?

☐ never ☐ rarely ☐ regularly ☐ frequently ☐ don't know / NA

ORGANIZATIONAL LEARNING

Suggested sources of information: program management, please validate with team members and stakeholders (if appropriate).

8.7 How often is existing/circulating information and knowledge collected and stored (basic learning)?

☐ never ☐ rarely ☐ regularly ☐ frequently ☐ don't know / NA

8.8 How often is collected information shared, discussed and acted upon at various levels within the organisation(s) (adaptive learning)?

☐ never ☐ rarely ☐ regularly ☐ frequently ☐ don't know / NA

8.9 How often is collected information shared, discussed and leads to change in fundamentals and objectives across all levels within the organisation(s) (generative learning)?

☐ never ☐ rarely ☐ regularly ☐ frequently ☐ don't know / NA

DIRECT ENVIRONMENT

Suggested sources of information: program management, team members and stakeholders (if appropriate).

8.10 How often is the direct environment of the initiative (involved stakeholders) **supportive for adaptive learning?** To score this question please consider in how far the direct environment accepts learning that focuses on correcting or improving existing procedures, processes, competences and technologies, as compared to expecting from you to never change procedures and processes and to never make mistakes.

☐ never ☐ rarely ☐ regularly ☐ frequently ☐ don't know / NA

8.11 **How often is the direct environment of the initiative** (involved stakeholders) **supportive for generative learning?** To score this question please consider in how far the general environment accepts learning that focuses on questioning the existing norms and that encourages looking beyond the existing situation, as compared to expecting from you to always adhere to existing norms and established paradigms.

☐ never ☐ rarely ☐ regularly ☐ frequently ☐ don't know / NA

GENERAL ENVIRONMENT

Suggested sources of information: program management, team members and stakeholders (if appropriate).

8.12 **How often is the general environment** (e.g. culture, economics, political situation) **of the initiative supportive for adaptive learning**? To score this question please consider in how far the general environment accepts learning that focuses on correcting or improving existing procedures, processes, competences and technologies, as compared to expecting from you to never change procedures and processes and to never make mistakes.

☐ never ☐ rarely ☐ regularly ☐ frequently ☐ don't know / NA

8.13 **How often is the general environment** (e.g. culture, economics, political situation) **of the initiative supportive for generative learning?** To score this question please consider in how far the general environment accepts learning that focuses on questioning the existing norms and that encourages looking beyond the existing situation, as compared to expecting from you to always adhere to existing norms and established paradigms.

☐ never ☐ rarely ☐ regularly ☐ frequently ☐ don't know / NA

9. Final considerations

**9.1 Do you have any immediate feedback for us?**

9.2 Would you like to name somebody as co-author in a potential publication on this data? In case you name someone else, it is necessary to provide the details of this person.

☐ Someone else ☐ Myself ☐ Nobody

Name of co-author:

Title of co-author:

Institutional affiliation of co-author:

Email contact of co-author:

Thank you for your time and patience!!!
